# Phylogenomics and Systematics of Overlooked Mesoamerican and South American Polyploid Broad-Leaved *Festuca* Grasses Differentiate *F.* sects. *Glabricarpae* and *Ruprechtia* and *F.* subgen. *Asperifolia, Erosiflorae, Mallopetalon* and *Coironhuecu* (*subgen. nov*.)

**DOI:** 10.3390/plants11172303

**Published:** 2022-09-02

**Authors:** María Fernanda Moreno-Aguilar, Luís. A. Inda, Aminael Sánchez-Rodríguez, Pilar Catalán, Itziar Arnelas

**Affiliations:** 1 Departamento de Ciencias Agrarias y del Medio Natural, Escuela Politécnica Superior de Huesca, Universidad de Zaragoza, C/Carretera de Cuarte Km 1, E-22071 Huesca, Spain; 2 Instituto Agroalimentario de Aragón, Universidad de Zaragoza, Centro de Investigación y Tecnología Agroalimentaria, E-50013 Zaragoza, Spain; 3 Departamento de Ciencias Biológicas y Agropecuarias, Universidad Técnica Particular de Loja, San Cayetano Alto s/n, Loja 1101608, Ecuador; 4 Grupo de Bioquímica, Biofísica y Biología Computacional (BIFI, UNIZAR), Unidad Asociada al CSIC, E-50059 Zaragoza, Spain

**Keywords:** allopolyploid speciation, Mexico–Central–South American broad-leaved *Festuca*, phylogeny, plastome, rDNA 45S and 5S genes, repeatome, taxonomy

## Abstract

Allopolyploidy is considered a driver of diversity in subtribe Loliinae. We investigate the evolution and systematics of the poorly studied Mesoamerican and South American polyploid broad-leaved *Festuca* L. species of uncertain origin and unclear taxonomy. A taxonomic study of seven diagnostic morphological traits was conducted on a representation of 22 species. Phylogenomic analyses were performed on a representation of these supraspecific taxa and all other Loliinae lineages using separate data from the entire plastome, nuclear rDNA 45S and 5S genes, and repetitive DNA elements. *F*. subgen. *Mallopetalon* falls within the fine-leaved (FL) Loliinae clade, whereas the remaining taxa are nested within the broad-leaved (BL) Loliinae clade forming two separate Mexico–Central–South American (MCSAI, MCSAII) lineages. MCSAI includes representatives of *F*. sect. *Glabricarpae* and *F*. subgen. *Asperifolia* plus *F. superba*, and MCSAII of *F*. subgen. *Erosiflorae* and *F*. sect. *Ruprechtia* plus *F. argentina*. MCSAII likely had a BL Leucopoa paternal ancestor, MCSAI and MCSAII a BL Meso-South American maternal ancestor, and Mallopetalon FL, American I–II ancestors. Plastome vs. nuclear topological discordances corroborated the hybrid allopolyploid origins of these taxa, some of which probably originated from Northern Hemisphere ancestors. The observed data indicate rapid reticulate radiations in the Central–South American subcontinent. Our systematic study supports the reclassification of some studied taxa in different supraspecific *Festuca* ranks.

## 1. Introduction

Despite considerable debate about the evolutionary fate of allopolyploids, alternatively viewed as drivers of biodiversity [1] or evolutionary dead ends [2], accumulating evidence suggests that hybridization and whole genome duplication (WGD) has been a preeminent evolutionary mechanism of speciation in the eukaryotic kingdom [3,4,5,6]. This is especially remarkable in seed and angiosperm plants, which are all considered descendants of paleopolyploid ancestors [7,8]. Allopolyploids are predominant in the grass family, accounting for 70% of the current species [9,10]. Despite genome duplication being considered generally irreversible in the short term [11], evidence suggests that the protograss whole genome duplication was likely followed by subsequent diploidizations that originated the respective ancestors of the Bambusoideae–Oryzoideae–Pooideae (BOP) and Panicoideae–Arundinoideae–Centothecoideae–Chloridoideae–Micrairoideae–Aristidoideae–Danthonioideae (PACCMAD) clades [12,13]. The evolutionary scenario of successive rounds of plant hybridizations and allopolyploidizations followed by the return to the diploid state [14] was also inferred for grasses. Grass mesopolyploids and neopolyploids were estimated to have originated some million years ago (Miocene–Pliocene) or during or after the Quaternary glaciations, respectively [11,15,16]. These allopolyploid speciation processes resulted in their current overwhelming representation within the grasses [10], with some genera consisting exclusively of hybrid allopolyploids (e.g., *Elymus* L., *Calamagrostis* Adans. [10,17]) and others containing a large number of them (e.g., *Festuca* L., [18]; *Poa* L., [19]). Molecular phylogenies have helped unravel the hybrid allopolyploid origin of some grass species for which their contrasting plastid vs. nuclear-based topologies have uncovered their respective maternal and paternal lineages [20], while their nuclear single-copy-genes-based topologies have uncovered phased alleles from the distinct progenitor lineages [21].

Subtribe Loliinae, one of the main lineages of the temperate Pooideae, is formed by the large paraphyletic genus *Festuca* and several closely-related genera nested within it [22,23,24,25,26,27]. Throughout the manuscript, the taxonomic names of *Festuca* are indicated in italics and the phylogenetic lineages of Loliinae in plain text. Phylogenetic analyses have consistently inferred two main clades within the subtribe, the broad-leaved (BL) and fine-leaved (FL) Loliinae, characterized by distinct genomic and phenotypic features [18,23,26,27]. *Festuca* contains approximately 600 species distributed worldwide, inhabiting cool seasonal regions of both hemispheres [18]. *Festuca*’s main center of diversity is the Holarctic region, which harbors nearly 500 species, including all known diploid species of the genus and different polyploids, ranging from tetraploids to dodecaploids [28]. It is also the inferred area for the origin of the BL and FL Loliinae ancestors, which later colonized the Southern Hemisphere according to DEC biogeographic models [25], a hypothesis consistent with the absence of Loliinae diploids in the Southern Hemisphere [18,27,29]. Nearly 80 species of *Festuca* occur in South America [30,31,32,33,34], an area that constitutes a secondary center of diversification of Loliinae and which was colonized several times from different regions [25,26]. Taxonomically, the *Festuca* species have been ranked into eleven subgenera according to the worldwide classification system of Alexeev [35,36,37,38,39,40,41,42,43,44]. Of these, the largest subgenus *Festuca*, which encompasses most of the fine-leaved taxa of both hemispheres, makes the bulk of the FL clade. It also includes the small subgenus *Helleria* E.B. Alexeev, also treated as a separate genus *Hellerochloa* Rauschert, and several other genera nested within [18,24,25,26]. The remaining nine *Festuca* subgenera, except the FL *Mallopetalon* (Döll) E.B. Alexeev, contain species of the BL clade, some of which have been also treated as separate genera. Two of them are native to the Old World (*Schedonorus* (P. Beauv.) Peterm., *Xanthochloa* (Krivot.) Tzvelev), five to the New World (*Asperifolia* E.B. Alexeev; *Subulatae* (Tzvelev) E.B. Alexeev, *Subuliflorae* E.B. Alexeev, *Erosiflorae* E.B. Alexeev, *Mallopetalon*) and two are native to both areas (*Leucopoa* (Griseb.) Hack., *Drymanthele* V.I. Krecz. & Bobrov). The BL clade also includes three additional separate genera nested within [22,25]. The species of the *Festuca* subgenera have been classified in different sections and subsections based on morphological traits ([18,23] and references therein). However, while some of these taxonomic ranks constitute robust lineages of both FL (Festuca (+Wangenheimia), Aulaxyper (+Vulpia 2x), Exaratae (+Loretia)) and BL (Schedonorus (+Lolium and Micropyropsis), Lojaconoa) Loliinae clades, others do not form monophyletic groups or mix with taxa from other *Festuca* ranks [24,25,26,27].

Although a large amount of biological and genomic resources has been generated for some economically important forage and grassland *Festuca* species (e.g., *F. pratensis* Huds., *F. arundinacea* Schreb.; [45]), other species of the genus have not been properly analyzed yet. Among the least phylogenetically and systematically studied, Loliinae species are polyploid taxa of six main broad-leaved *Festuca* groups (*Festuca* subgenera *Asperifolia*, *Drymanthele* (sect. *Ruprechtia* E.B. Alexeev), *Erosiflorae*, *Mallopetalon*, *Subulifolia* (sect. *Glabricarpae* E.B. Alexeev), and *F. argentina* (Speg.) Parodi), endemic to Mexico, Central America or South America, some of which constitute the basal-most BL lineages but have uncertain taxonomic adscriptions and evolutionary circumscriptions [24,25,26,27]. All of them, except *F. argentina*, include tall fescues that show extravaginal (or mixed) innovations, flat leaves, and open and lax panicles. In a series of successive taxonomic studies, Alexeev described *Festuca* sect. *Ruprechtia* (type specimen *F. amplissima* Rupr.) [37], *F*. subgen. *Asperifolia* (type specimen *F. lugens* (E. Fourn.) Hitchc. ex Hern.-Xol) [38], *F*. sect. *Glabricarpae* (type specimen *F. breviglumis* Swallen) [43], *F*. subgen. *Mallopetalon* (type specimen *F. fimbriata* Nees) [44] and *F*. subgen. *Erosiflorae* (type specimen *F. quadridentata* Kunth) [42] based on the types of innovation leaves, ligules and lemmas, and the presence or absence of ciliate lodicles and of plant and ovary induments. *Festuca argentina*, initially assigned to *F*. subgenus *Festuca* [46], was also considered close to *F*. subgen. *Mallopetalon* [47]; however, it is morphologically different [32] and phylogenetically divergent [24,25] from both taxa. The five subgeneric and sectional *Festuca* ranks described by Alexeev were expanded with other close species described from Mesoamerica and South America by the same or later authors (Table 1). Stančík and Peterson [31] and Stančík and Renvoize [48] extended the concept of *F*. subgen. *Erosiflorae sensu* Alexeev including new broad-leaved South American *Festuca* species within this taxon (e.g., *F. superba* Parodi ex Türpe, *F. venezuelana* Stančík) and transferring taxa from *F*. sect. *Glabricarpae* (e.g., *F. steinbachii* E.B. Alexeev) to it but without strong morphological or phylogenetic arguments.

Despite the importance of previous taxonomic work, the broad-leaved species belonging to these groups have been little studied, and the morphological characters used to delimit their taxonomic ranks remain poorly understood. The high uncertainty about the taxonomic circumscriptions and the evolutionary placements of the five Mesoamerican–South American taxonomic *Festuca* ranks described by Alexeev plus *F. argentina* are of high interest as these polyploid taxa may constitute some of the ancestral lineages of the broad-leaved Loliinae [25,27]. Therefore, the objectives of our study were to: (i) evaluate past classifications and identify diagnostic morphological characters that could serve to circumscribe the taxa; (ii) use genomic data to reconstruct a solid phylogenomic framework to reveal their evolutionary position within the phylogeny of subtribe Loliinae; (iii) detect the putative maternal and paternal origins of these lineages using plastome-based vs. nuclear-based phylogenies; and (iv) propose a reclassification for these taxa based on morphological and molecular evidence.

## 2. Results

### 2.1. Taxonomic Study

**Figure 1:**
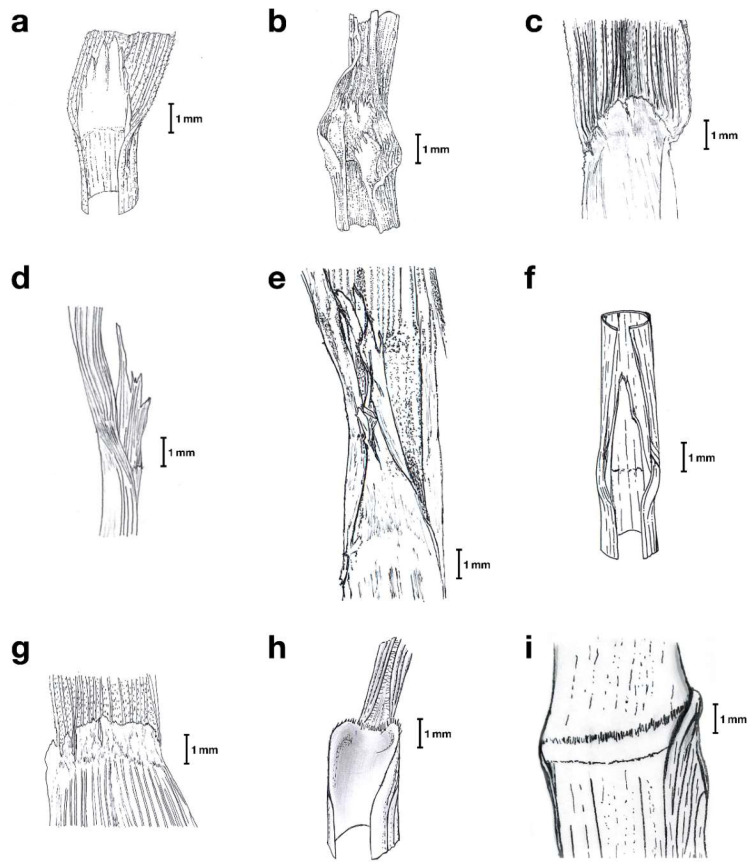
Ligule shape of representative species of Mesoamerican and South American broad-leaved *Festuca* taxa analyzed morphologically in this study. *F*. subgen. *Subulatae* sect. *Glabricarpae*: *F. venezuelana* (**a**); *F*. subgen. *Drymanthele s. l*.: *F. superba* (**b**); *F*. subgen. *Subulatae* sect. *Glabricarpae*: *F. breviglumis* (**c**); *F*. subgen. *Asperifolia*: *F. asperella* (**d**); *F*. subgen. *Erosiflorae*: *F. quadridentata* (**e**,**f**); *F*. subgen. *Drymanthele* sect. *Ruprechtia*: *F. amplissima* (**g**); *F.* subgen. *Coironhuecu* (*subgen. nov*.): *F. argentina* (**h**); *F*. subgen. *Mallopetalon*: *F. fimbriata* (**i**). Drawings by José Alfredo Hidalgo-Salazar (**a**–**h**) and María Fernanda Moreno-Aguilar (**i**). ((**a**,**f**): modified from Stančík and Peterson [31]; (**b**): modified from Türpe [49]; (**c**): Peterson P. M. and Rosales O. 16117, US-3524155; (**d**): Dziekanowski et al., 2022, MO-2107299 (isotype); (**e**): Laegaard S. 55567, AAU; (**g**): Peterson P. M. and Herrera-Arrieda Y. 16150, US-3524157; (**h**): modified from Ospina [34]; (**i**): Kostling M. UZ 498.08).

The analysis of seven morphological traits used by Alexeev to diagnose the studied *Festuca* subgeneric and sectional ranks (plant habit, type of innovation leaves, ligule type and apex shape, leaf-blade type, inflorescence type, lemma apex shape, ovary tip hairiness) plus an additional reproductive trait (monoecy vs. dioecy) (Table 1, Figure 1 and Supplementary Figure S1) on the species classified within these ranks allowed us to identify the taxa proposed by Alexeev and describe a new supraspecific taxon of *Festuca*.

Species included in *F*. subgen *Erosiflorae sensu* Alexeev [42] are characterized by their monoecy, rhizomatous, tussocked or mixed habit, displaying extravaginal and intravaginal innovation leaves, a long membranous ligule with erose or lacerate apex, flat leaf blades, partially involuted at apex, erect panicles (without nutant branches), unawned dentate or entire lemma apex, and glabrescent ovary tip. These features are present in the type species *F. quadridentata*, endemic from the Ecuadorean paramos, and in two other species distributed in the northern Andes, *F. dichoclada* Pilg. and *F. horridula* Pilg., incorporated into this subgenus by Alexeev [42] (Table 1 and Supplementary File S1; Figure 1 and Supplementary Figure S1). Stančík and Peterson [31] and Stančík and Renvoize [48] expanded the circumscription of *F*. subgen. *Erosiflorae* to six new South American species of which two fulfilled all the main diagnostic characteristics proposed by Alexeev (*F*. *carrascana* Stančík & Renvoize, *F. chuquisacae* Stančík & Renvoize)*,* one differed from them due to its shortly awned lemma (*F. urubambana* Stančík), another due to its partially nutant panicles and awned lemma (*F. venezuelana*), the fourth for its densely hairy ovary tip, shorter hilum and hyaline ligule with dentate apex (*F. superba*), and the fifth for its short ligule with ciliate apex, nutant panicles and awned lemma (*F. steinbachii*) (Table 1 and Supplementary File S1). Species classified within *F*. subgen *Drymanthele* sect. *Ruprechtia sensu* Alexeev [37] differentiated from those of *F*. subgen. *Erosiflorae* in their short non-membranous ligule with truncate and shortly ciliate apex and in their entire non-dentate lemma apex. It includes the type species *F. amplissima*, distributed in Mexico, Central America and northern South America, and two additional species endemic to Mexico, *F. jaliscana* E.B. Alexeev and *F. valdesii* Gonz.-Led. & S.D. Koch. (Table 1, Supplementary File S1 and Figure 1). The species classified within *F*. subgen. *Subulatae* sect. *Glabricarpae* [43] are separated from *F*. subgen. *Erosiflorae* in their shorter ligules with truncate or rounded and lacerate or dentate apex, nutant panicles or panicle branches, and their entire or bifid and awned lemma apex, and from *F*. subgen. *Drymanthele* sect. *Ruprechtia* in their membranous ligule, nutant panicles and awned lemma apex (Table 1 and Figure 1). Alexeev classified within this section the species type *F. breviglumis*, distributed in Central America and Mexico, and other Mesoamerican and northern South American species, *F. chiriquensis* Swallen, *F. caldasii* (Kunth) Kunth and *F. steinbachii* [41,43]. Stančík and Peterson [50] added to *F*. sect. *Glabricarpae* the North Andean species *F. dentiflora* E.B. Alexeev ex Stančík & P.M. Peterson and *F. woodii* Stančík, which matched the sectional diagnostic features except for the sparsely hairy ovary tip of *F. woodii* (Table 1 and Supplementary File S1). The species classified in *F*. subgen. *Asperifolia sensu* Alexeev [38] departed from the previous taxa in their densely tussocked habit, medium-length membranous ligule with truncate or slightly rounded and dentate or lacerate apex, bifid and short-awned (or awned) lemma apex, and glabrous to sparsely hispid ovary tip. The subgenus includes the type species *F. lugens*, endemic to Mexico and Central America, and other species endemic to Mexico, *F. asperella* E.B. Alexeev and *F. tancitaroensis* Gonz.-Led. & S.D. Koch (Table 1, Supplementary File S1 and Figure 1). *F*. subgen. *Mallopetalon* was described by Alexeev [44] based solely on the type species *F. fimbriata*, which shows some diagnostic traits shared with one or the other previously described taxa, such as the possession of a long rhizomatous habit, a short membranous ligule with erose and ciliate apex, and erect multispiculate panicle, but differentiated from all of them in its fimbriated lodicles, scarious, rolled and fimbriated lemma apex, and densely hairy ovary tip (Table 1, Supplementary File S1 and Figure 1).

We have examined taxonomically and phylogenomically two other species evolutionarily close to the five supraspecific *Festuca* lineages mentioned above. *Festuca superba*, a narrow endemic species from northwestern Argentina, was classified within the *F*. subgen. *Erosiflorae* by Stančík and Renvoize [48] based on general gross morphological traits shared with this taxon. However, it separates from the species of this rank and from the other taxa on the basis of its broad flat leaves with subconvolute vernation, multispiculate inflorescences with flexuous branches, muticous lemma apex and densely hairy ovary tip (Table 1 and Supplementary File S1; Figure 1 and Supplementary Figure S1). *Festuca argentina*, endemic to Patagonia and the southern Andes, is the most phenotypically distinct species of all taxa analyzed. It has been attributed to fine-leaved *F*. subgen. *Festuca* by some authors [46] due to its caespitose habit and plicate and junciform leaves (Table 1 and Supplementary File S1; Figure 1 and Supplementary Figure S1). However, *F. argentina* shows unique traits, such as dioecy, a narrowly contracted lanceolate panicle and a sparsely hispid ovary tip (Table 1).

### 2.2. Phylogenomic Analyses

Phylogenomic analyses of a selection of 11 *Festuca* species, representing the five supraspecific *Festuca* ranks of Alexeev and the two close phylogenetic taxa (Table 1), plus 23 additional Loliinae species, representing the 20 evolutionary lineages detected within the subtribe [25,26], were performed using assembled nuclear rDNA 35S and IGS, nuclear rDNA 5S and plastomes retrieved from genome skimming sequencing data (Table 2). New genome skims obtained 10 species, including three species not investigated molecularly before (*F. chiriquensis, F. horridula*, *F. venezuelana*) and seven species characterized only for a few loci (*F. argentina, F. asperella, F. breviglumis, F. dichoclada, F. gautieri* (Hack.) K. Richt., *F. kingii* (S. Watson) Cassidy, *F. valdesii*), along with genome skimming data on five species of the supraspecific *Festuca* taxa under study (*F. amplissima*, *F. caldasii*, *F. fimbriata, F. quadridentata*, *F. superba*) and 21 species of other Loliinae lineages and two outgroups obtained in previous works [26,27] were used in the analyses. Additionally, nuclear repetitive DNA element frequency data, extracted from the genome skimming data, were used to investigate the evolutionary placement of representative species of the taxa under study within a Loliinae-wide repeatome phylogenetic framework and to compare its topology with those obtained from the plastome and rDNA sequence data sets. Although polyploidy can have a large impact on phylogenies, haploid plastomes are maternally inherited in Loliinae and are not sensitive to ploidy level. In contrast, rDNA genes may be affected by convergent evolution to one or another subgenome and/or by gene loss, or may be missed by genome skimming approaches if some of the subgenomic ribotypes are present at low frequencies in the nuclear genome. The subgenomic repetitive elements may be balanced or may have dominant/submissive contents between subgenomes, although this could not be clarified with genome skimming data alone. However, all these approaches together allowed us to infer the evolutionary history of the species under study.

Genome skimming data from newly sequenced samples ranged from 5683 (*F. asperella*) to 32,808 (*F. horridula*) million Illumina pair-end (PE) reads (Table 2). The sequences of the assembled nuclear rDNA 45S region were split into a transcribed 35S cistron data set and an untranscribed intergenic spacer (IGS) data set. The length of the 35S cistron sequence ranged from 6521 (*F. kingii*) to 6532 bp (*F. chiriquensis*), with a total length of 6589 bp in the multiple sequence alignment (MSA) (894 variable sites, 381 parsimony informative sites). This region showed a conserved structure along its aligned transcriptional unit, composed of the 5′-external transcribed spacer (ETS) (~715 bp), the 18S gene (1818 bp), the internal transcribed spacers and the 5.8S gene (ITS1-5.8S-ITS2) (577 bp), and the 25S gene (3392 bp), which had similar average lengths in the samples studied. The highly variable IGS region, studied for the first time in Loliinae, ranged from 977 (*F. pratensis*) to 1992 bp (*F. gracillima* Hook. f.), producing an MSA 2496 bp in length (1439, 919). The newly assembled sequences of the nuclear rDNA 5S region ranged from 298 bp (*F. kingii, F. valdesii*) to 319 bp (*F. gautieri*). The 5S region consisted of a conserved 5S gene (120 bp in all species) and a 563 bp intergenic variable spacer (IGS) in the MSA (158, 109). The newly assembled plastomes ranged from 131,438 bp (*F. superba*) to 133,638 bp (*F. chiriquensis*), matching the plastome length values obtained in previous studies [26,27] for the respective Loliinae FL and BL clades. Most of the newly assembled plastomes showed good read coverage (>40×) except *F. breviglumis* and *F. valdesii*, which had lower read coverage (13×–26×). The MSA of the plastomes was 134,265 bp in length (14,397, 4776). Newly obtained sequences from each data set were deposited in GenBank under accession codes OP120917-OP120926 (35S), OP158132-OP158167 (IGS), OP142676-OP142686 (5S), SAMN30029287-SAMN30029296 (plastomes) (SRA data under BioProject PRJNA863311) (Table 2).

**Figure 2:**
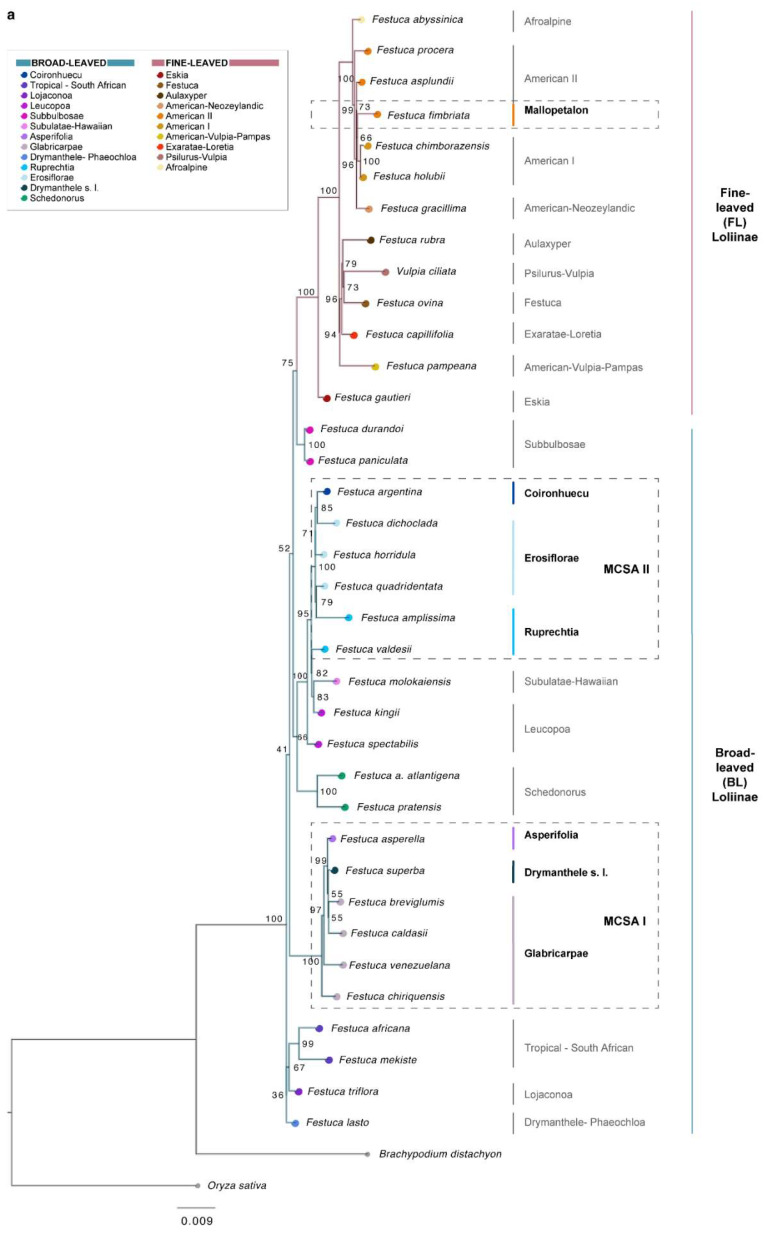

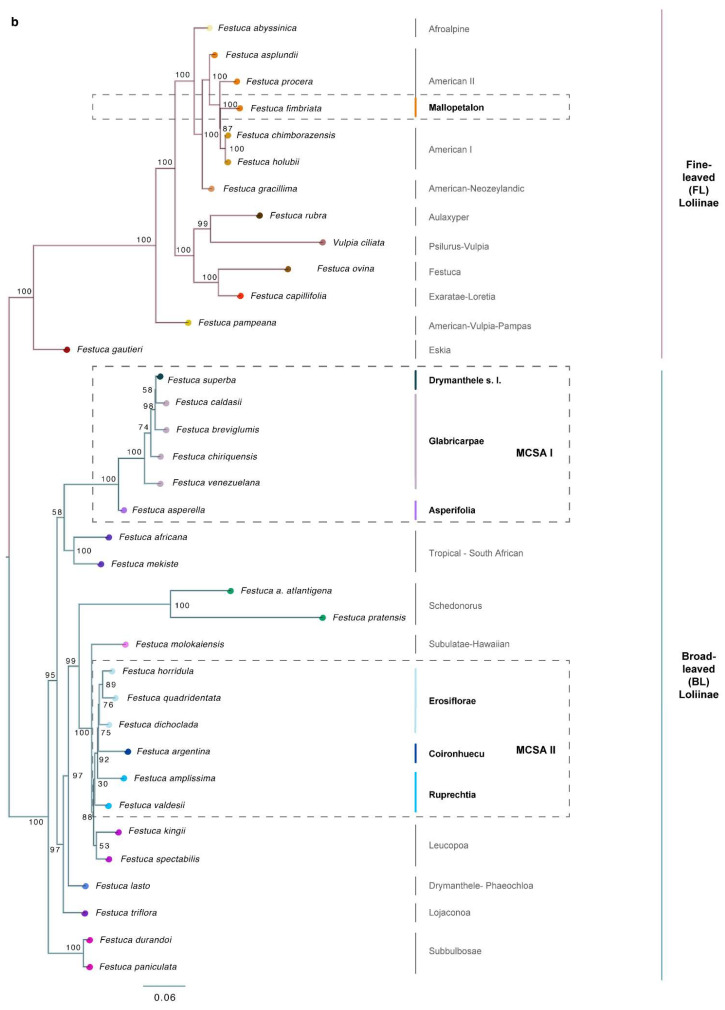

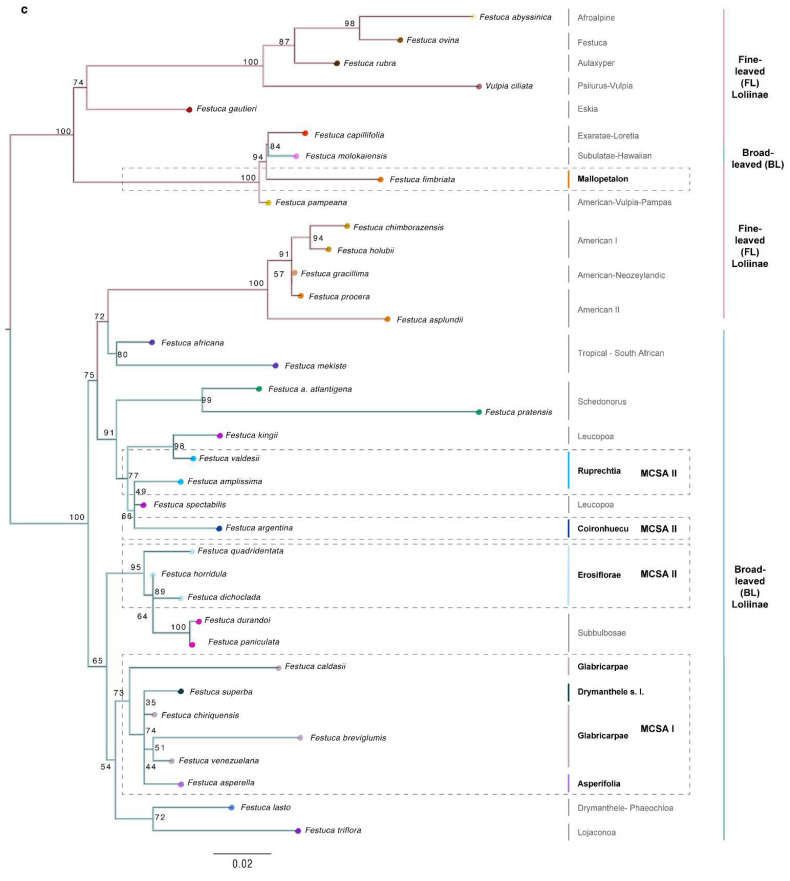

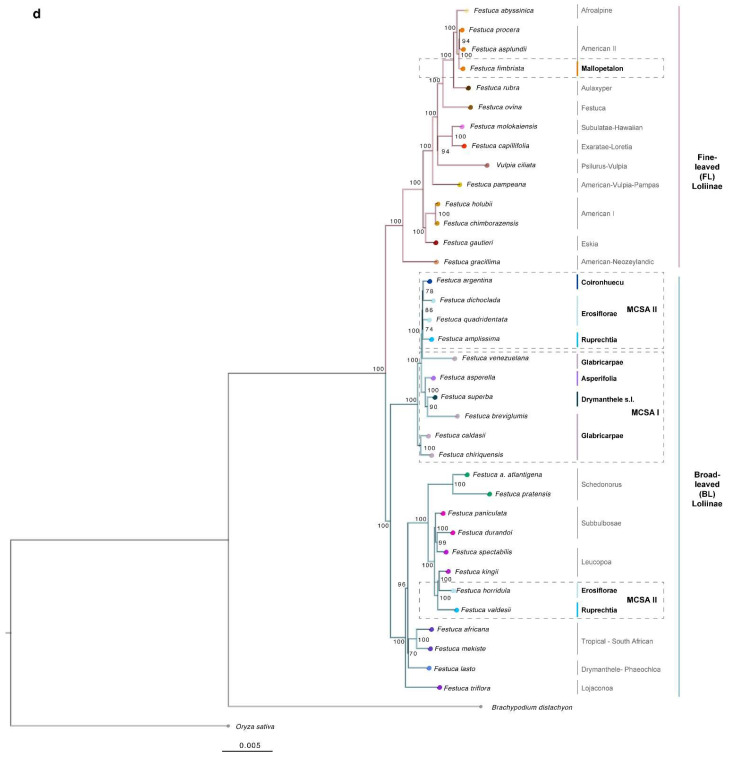
Maximum likelihood phylogenomic trees of the Mesoamerican and South American broad-leaved *Festuca* taxa studied and other representative species of the broad-leaved (BL) and fine-leaved (FL) Loliinae lineages. (**a**) Nuclear rDNA 35S cistron tree. (**b**) Nuclear rDNA (45S) IGS tree. (**c**) Nuclear rDNA 5S tree. (**d**) Plastome tree. Mexico–Central–South American (MCSAI, MCSAII) and Mallopetalon groups are indicated by discontinuous-line rectangles. Ultrafast bootstrap support values are indicated on branches. *Oryza sativa* and *Brachypodium distachyon* outgroups were used to root the trees except for the IGS and 5S trees that were rooted at midpoint. Color codes of Loliinae lineages are indicated in the chart of (**a**). Scale bars: number of mutations per site.

The 35S maximum likelihood (ML) phylogenetic tree recovered the expected topology for the Loliinae as previously presented by Moreno-Aguilar et al. [26], consisting of a fully supported FL clade and a series of strongly to weakly supported basal paraphyletic BL lineages (Figure 2a). In this tree, *F. fimbriata* (Mallopetalon lineage) was nested within a strongly supported FL American I–American II clade, whereas the remaining species under study fell into two separate BL groups. Representative species of *F*. sect. *Glabricarpae* (*F. breviglumis, F. cladasii, F. chiriquensis*) and *F*. subgen. *Asperifolia* (*F. asperella*), together with *F. venezuelana* and *F. superba*, formed a robust Mexico–Central–South American I (MCSA I) clade, while the representative species of *F*. sect. *Erosiflorae* (*F. dichloclada, F. horridula, F. quadridentata*), *F*. sect. *Ruprechtia* (*F. amplissima*, *F. valdesii*) and *F. argentina* formed a Mexico–Central–South American II (MCSA II) group integrated into a robust clade that also included representatives of Leucopoa (*F. kingii*, *F. spectabilis* Bertol.) and Subulatae-Hawaiian (*F. molokaiensis* Soreng, P.M. Peterson & Catalán) (Figure 2a). The (45S) IGS ML tree, first computed for the Loliinae in the present study, showed two fully supported FL and BL sister clades (Figure 2b). *F. fimbriata* (Mallopetalon) was also nested within a robust FL American I–American II clade, whereas the other taxa fell within the BL clade. The robust MCSA I clade (Glabricarpae–Asperifolia–*F. superba*–*F. venezuelana*) was resolved as a sister to the also robust tropical–South African clade, although this relationship was weakly supported, and the strongly supported MCSA II clade (Erosiflorae–Ruprechtia–*F. argentina*) was resolved as a sister to a weakly supported Leucopoa clade, although this relationship was well supported (Figure 2b). The 5S ML tree was congruent with the 45S (35S, IGS) ML trees for some but not all lineages (Figure 2c). The 5S-based tree topology also recovered a relatively well supported MCSAI clade, which was resolved as a sister to an Old World Drymanthele/Lojaconoa clade, although this relationship was poorly supported. In contrast, the MCSAII group split into two separate lineages on this tree; in one of them, Erosiflorae species formed a strongly supported clade together with Old World Subbulbosae species, and in the second lineage Ruprechtia and *F. argentina* species joined in a relatively well supported clade together with American and European Leucopoa species. In this 5S-based topology, *F. fimbriata* (Mallopetalon) was also nested within the FL Loliinae clade but close to representative species of American Pampas, Subulatae-Hawaiian and Exaratae lineages and not to those of American II, American I and American–Neozeylandic lineages, which formed a nested group within the BL Loliinae clade (Figure 2c). The plastome-based ML tree also recovered two fully supported FL and BL sister Loliinae clades (Figure 2d). In this matrilineal phylogeny, *F. fimbriata* was nested within a fully supported FL American II lineage, and the remaining species under study within different groups of the BL clade. Species from the MCSAI (all) and MCSAII (pro parte) groups formed a clade, sister to another clade that included two species from the MSCAII group and representatives of the remaining BL lineages, with all these relationships showing full support. Within the MCSA superclade, Glabricarpae, Asperella and *F. superba* (MCSAI group) species were resolved as basal paraphyletic lineages, while *F. venezuelana* formed a fully supported clade with most elements of the more recently evolved and well supported MCSAII pro parte clade. The two species of the MCSAII group that departed from the MCSA superclade, *F. horridula* (Erosiflorae) and *F. valdesii* (Ruprechtia), formed a fully supported subclade together with American *F. kingii* (Leucopoa); this subclade, in turn, joined other Eurasian species of Leucopoa and of Subbulbosae in a fully supported lineage (Figure 2d). To account for potential incomplete lineage sorting, we performed parallel phylogenetic analyses with the same data sets but modeling the coalescence process using the Singular Value Decomposition quartets (SVDq) approach implemented in Paup *, which combines quartet trees into a species tree. Since the topologies of the 35S, IGS, 5S and plastome SVDq trees (Supplementary Figure S2a–d) were the same as those of the ML trees, or recovered similar lineages, only the latter were described. The (45S) IGS ML tree was used to map the diagnostic morphological traits of the supraspecific *Festuca* ranks under study on its branches (Supplementary Figure S3).

**Figure 3:**
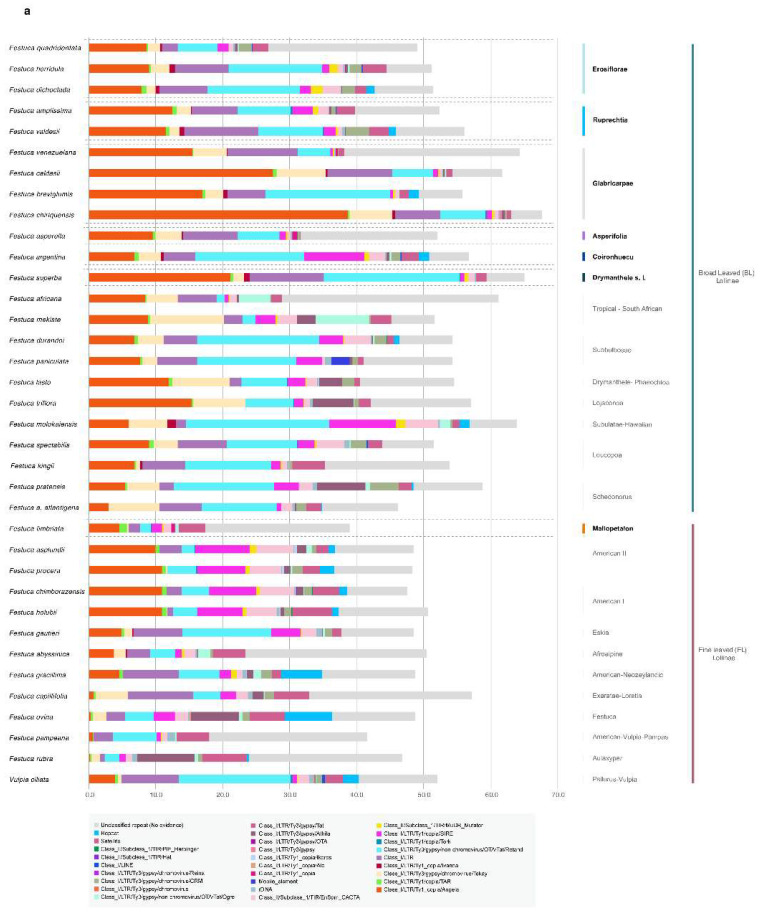

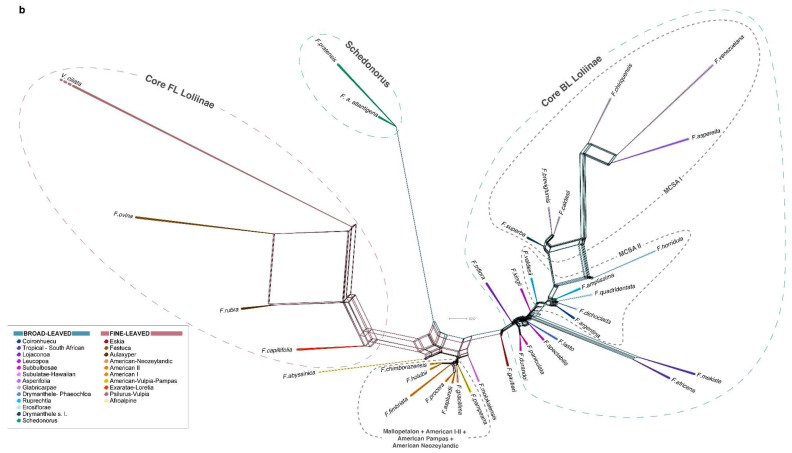
(**a**) Histograms of repeat contents per holoploid genome (1C) retrieved from the individual Repeat Explorer 2 (RE2) analyses of the studied Mesoamerican and South American broad-leaved *Festuca* taxa and other Loliinae samples. Color codes for repeat types are indicated in the chart. (**b**) Phylogenetic network based on standardized repeat data sets retrieved from the comparative RE analysis and constructed from distance-based NJ trees computed with pairwise Euclidean distances between samples. Core BL, core FL, Schedonorus, Mexico–Central–South American (MCSAI, MCSAII), and Mallopetalon + other American Loliinae groups are surrounded by dashed lines. Color codes of Loliinae lineages are indicated in the chart.

The annotated nuclear repetitive elements found by Repeat Explorer 2 (RE2) in the individual analysis of the newly sequenced samples (Supplementary Table S1 and Figure 3a) were consistent with data from a previous study of representative groups of Loliinae [27]. Repeat elements contributed to large proportions of the MCSAI and MCSAII haploid genomes (mean 56.8%; ranging from 49.0% (*F. quadridentata*) to 67.5% (*F. chiriquensis*) (Supplementary Table S1). Interestingly, *F. fimbriata* (Mallopetalon) showed the lowest percentage of repeatomes (38.8%) among the studied species, differing from the relatively high values shown by the American II and American I species (Supplementary Table S1) but being close to the observed values in other high-polyploid Loliinae species (e.g., *F. arundinacea*; [27]). LTR-Copia and LTR-Gypsy retrotransposons represented the major fractions of the repeatomes followed by Class II TIR-transposons and satellite repeats in the newly studied genomes. Of them, LTR-Copia Angela and LTR-Gypsy Retand elements were the most frequent repeat families in all the BL species studied (Supplementary Table S1; Figure 3a). Glabricarpae and *F. superba* showed high coverages of Angela elements, and Erosiflorae, Ruprechtia, *F. breviglumis* (Glabricarpae), *F. argentina* and *F. superba* of Retand elements. *F. fimbriata* had a low coverage of Retand elements, as in some FL American II species (e.g., *F. asplundii* E.B. Alexeev), although unlike the American II and American I species, it showed a much lower coverage of Angela elements (Supplementary Table S1; Figure 3a). A total of 37 top repeat clusters, annotated by RE2 in the comparative analysis of all 36 Loliinae genomes, were used to construct a combined phylogenetic network from the respective distance-based Neighbor-Joining (NJ) trees. The topology of the unrooted Loliinae repeatome network showed the divergence of three main groups, BL (core), FL (core) and Schedonorus lineages, with representatives of the American I, American II, American Pampas, American–Neozeylandic, Subulatae-Hawaiian and Afroalpine lineages occupying an intermediate position between the core FL and BL subnetworks (Figure 3b). The MCSAI and MCSAII species clustered into their respective divergent groups and formed a large MCSA supergroup within the core BL subnetwork; North American *F. kingii* (Leucopoa) was resolved as the closest relative of this MCSA supergroup (Figure 3b). *F. fimbriata* (Mallopetalon) fell within the expanded FL group in this repeatome-based network, nesting in an intermediate position between the American II and American I lineages (Figure 3b). The representative species of fine-leaved *F*. sect. *Eskia*, *F. gautieri*, clustered closer to the BL core group than the FL core group, as previously observed for other species in this section (*F. eskia* Ramond ex DC. [27]).

## 3. Discussion

### 3.1. Evolutionary History of Allopolyploid Broad-Leaved Mexico–Central–South American Festuca Lineages (Erosiflorae, Ruprechtia, Glabricarpae, Asperella, Mallopetalon, F. argentina, F. superba)

Our taxonomic and phylogenomic analyses of overlooked Mexico–Central–South American broad-leaved *Festuca* lineages have been instrumental in unravelling the origins and systematics of the seven Loliinae groups studied (Figure 1, Figure 2 and Figure 3 and Supplementary Figure S1, Table 1 and Table 2 and Supplementary Table S1). Our results indicate that *F. fimbriata* (*F*. subgen. *Mallopetalon*) originated from ancestors of FL Loliinae, while species in the other six groups derived from ancestors of BL Loliinae (Figure 2 and Figure 3). This highly divergent evolutionary position of *F. fimbriata* with respect to its morphologically close congeners might be associated with the recent reticulated radiation of polyploid South American *Festuca* species within the FL clade from the early Pliocene to the Pleistocene [25,26]. The “broad-leaved syndromes” that *F. fimbriata* presents in its habit, innovation leaves and inflorescence (Table 1 and Supplementary Figure S3) are also shared by other robust “BL-type” *Festuca* species, which have also originated within the large and phenotypically variable American II (e.g., *F. peruviana* Infantes) and American I (e.g., *F. purpurascens* Banks & Sol. ex Hook. f.) “fine-leaved” lineages [25,26]. However, some of the private morphological features characteristic of *F. fimbriata*, such as the possession of fimbriated lodicles and lemma apex (Table 1), support its classification in the separate *F*. subgenus *Mallopetalon* [44]. *F. fimbriata* is also unique in its adaptation to an exceptional ecological habitat for Loliinae, the flooded swamps of southern South America [32,47]. This allohexaploid species (Table 2) likely originated from an American II maternal ancestor (plastome tree; Figure 2d) and an American I paternal ancestor (nuclear 35S, IGS trees; Figure 2a,b). Its allohexaploidy is corroborated by its asymmetric and heterogeneous karyotype [47], characteristic of polyploid hybrid plants derived from progenitor species with different chromosomal complements [51,52]. Its relatively low percentage of repetitive elements per haploid genome (Supplementary Table S1 and Figure 3a) agrees with those observed in other allohexaploid species of *Festuca* [27]. Despite some morphological similarities with *F. argentina* (Table 1), both species occupy widely divergent positions in opposite Loliinae lineages (the robust *F. fimbriata* nested within the FL clade and the more slender *F. argentina* within the BL clade), as shown in the nuclear, plastome and repeatome phylogenies (Figure 2 and Figure 3b), thus ruling out any close relationship between them and confirming the great plasticity of some of the morphological traits used to separate *Festuca* taxa [23].

Species from the other six broad-leaved Loliinae groups studied fell into two separate BL lineages (MCSAI, MCSAII) in the 35S, IGS, 5S (MCSAI) and repeatome-based nuclear phylogenies (Figure 2a,b and Figure 3b), while in the plastome-based phylogeny, almost all species of both groups shared a common ancestor (Figure 2d). The relatively more ancestral MCSAI clade includes representative species of *F*. subgen. *Asperifolia* (*F. asperella*) and *F*. sect. *Glabricarpae* (*F. breviglumis, F. caldasii, F. chiriquensis*) plus *F. venezuelana* and *F. superba* (Figure 2 and Figure 3). Asperifolia and Glabricarpae taxa share morphological features such as the possession of a membranous ligule with a truncate apex and awned lemma (except in *F. tancitaroensis*), while they differ in their erect (Asperifolia) vs. nutant (Glabricarpae) panicles (Table 1, Figure 1 and Supplementary Figure S3). *F. venezuelana* and *F. superba* were classified by Stančík and Renvoize [48] within *F*. subgen. *Erosiflorae*. However, *F. venezuelana* is morphologically closer to Glabricarpae than to Erosiflorae for the diagnostic traits examined (e.g., nutant panicle, awned lemma; Table 1), which together with its phylogenetic placement within the Glabricarpae lineage (Figure 2, Figure 3 and Supplementary Figure S3), supports its taxonomic transference to *F*. sect. *Glabricarpae*. *F. superba* is morphologically separated from the Erosiflorae and the Glabricarpae–Asperifolia groups (Table 1), although its taxonomic classification is still unclear (see comments below). The expanded Glabricarpae group, therefore, shows a relatively consistent evolutionary history, although it is made up of paraphyletic lineages in most trees and the nuclear phylogenetic network (Figure 2a–c and Figure 3), with Asperifolia and *F. superba* nested in its clade. Glabricarpae is also reconstructed into a series of basal and subbasal lineages in the MCSA superclade of the plastome tree (Figure 2d).

The relatively more recently evolved MCSAII clade integrates representative species of *F*. subgen. *Erosiflorae* sensu Alexeev (*F. dichoclada, F. horridula, F. quadridentata*) and *F*. sect. *Ruprechtia* (*F. amplissima, F. valdesii*) plus *F. argentina* (Figure 2 and Figure 3). The Erosiflorae and Ruprechtia taxa share common morphological traits, both presenting erect panicles, unawned lemmas and mostly glabrous ovary tips, while differing in the overall long erose or lacerated membranous ligule with an acute and dentate lemma apex of Erosiflorae vs. the overall short non-membranous ligule with a truncate and non-dentate lemma apex of Ruprechtia (Table 1 and Figure 1). In the IGS nuclear phylogeny, the three species of Erosiflorae are reconstructed as a monophyletic group (Figure 2b and Supplementary Figure S3), reinforcing the classic taxonomic circumscription of this taxonomic rank proposed by Alexeev [42]. Although not studied genomically, other species included within *F*. subgen. *Erosiflorae* by Stančík and Renvoize [48], such as *F. steinbachii*, did not fit the diagnostic traits of Erosiflorae but rather those of its earlier *F*. sect. *Glabricarpae* classification [41], as this species has nutant panicles, a short ligule with a truncate and ciliate apex, and an awned lemma (Table 1). Therefore, the taxonomic circumscription proposed by Stančík and Renvoize [48] for *F*. subgen. *Erosiflorae* has been shown to be morphologically and phylogenetically artificial. In the nuclear 45S and 5S and repeatome network phylogenies, the two Ruprechtia species studied are resolved as paraphyletic, although they are closely related to each other (Figure 2a–c and Figure 3b). Of these, *F. amplissima* is more morphologically and phylogenetically related to Erosiflorae + *F. argentina* than *F. valdesii* (Table 1, Figure 2a–c and Figure 3b and Supplementary Figure S3). *Festuca valdesii*, classified within *F*. sect. *Ruprechtia* by González-Ledesma et al. [53], differs from the two species assigned to the section by Alexeev (*F. amplissima*, *F. jaliscana*) in its non-rhizomatous caespitose habit, longer membranous ligule with a truncate and short ciliate apex and hispid ovary tip (Table 1), raising doubts about its definitive systematic classification. Although deeply nested within the MCSAII clade in all nuclear and plastome-based phylogenies (Figure 2 and Figure 3b), *F. argentina* differs morphologically from Erosiflorae and Ruprechtia, as well as from the MCSAI Asperifolia and Glabricarpae taxa (Table 1, Figure 1 and Supplementary Figure S3), and therefore deserves an independent taxonomic classification (see comments below). Interestingly, in the nuclear rDNA 35S and IGS phylogenies, the Erosiflorae, Ruprechtia and *F. argentina* lineages fall into a larger, fully supported clade that also includes closely-related species of the *F*. subgen. *Leucopoa* (*F. kingii*, *F. spectabilis*) and Subulatae-Hawaiian (*F. molokaiensis*) lineages (Figure 2a,b), while in the plastome phylogeny, one species of Erosiflorae (*F. horridula*) and one species of Ruprechtia (*F. valdesii*) split from the MCSA superclade and fell within a separate BL lineage, nesting with the North American Leucopoa *F. kingii* in a strongly supported clade (Figure 2d). The closeness of the MCSAII group to *F. kingii* was also recovered in the repeatome network (Figure 3b).

The different topological positions of the MCSAI and MCSAII lineages in the nuclear vs. plastome trees and in the repeatome network (Figure 2 and Figure 3b) confirm the putative hybrid origins of these polyploid BL *Festuca* species [25,27]. The origins of these allopolyploids could be partially unraveled from our phylogenomic data. Thus, the MSCAII lineages (Erosiflorae, Ruprechtia, *F. argentina*), probably derived from a *Leucopoa* ancestor, which likely acted as the paternal parent for most of these species (nuclear 35S and IGS trees; Figure 2a,b), and from an unknown maternal MCSA parent (plastome tree; Figure 2d). Furthermore, *F. horridula* (Erosiflorae) and *F. valdesii* (Ruprechtia) likely had both paternal and maternal Leucopoa-type parents (Figure 2a,b,d). However, the origins of the MCSAI lineages (Glabricarpae, Asperifolia, *F. superba*) are less clear. The nuclear topologies do not retrieve strongly supported relationships of these slightly older MCSA lineages with any of the remaining BL lineages (Figure 2a–c), while the plastome phylogeny indicates that the MCSAI group shared the same maternal parent as most of the MCSAII taxa (Figure 2d). This would imply three potential colonizations of Eurasian and/or North American *Festuca* lineages to Central and South America. One of them probably contributed as the maternal parent of most of the MCSAI and MCSAII species and the other two probably contributed as respective paternal parents of the MCSAI and MCSAII (Leucopoa-type) groups. This hypothesis agrees with the proposed DEC biogeographic models for colonizing ancestral BL *Festuca* lineages from the Northern Hemisphere to Mesoamerica and South America [25,27]. The MCSAI and MCSAII nuclear and plastome phylogenies show a trend of more ancestral Mesoamerican and northern South American lineages and more recently evolved southern South American lineages within both clades (Figure 2a–d), which support the North-to-South stepwise colonization pattern proposed for the American *Festuca* ancestors [25]. The absence of diploid species of *Festuca* in these regions and throughout the southern hemisphere [18,29] allows us to speculate that the ancestral colonizers that originated the MCSAI and MCSAII lineages may have been polyploids; however, the lack of supported sister relatives precludes the inference of their putative ploidy levels. The studied species also comply with the observed trend of increasing ploidy level with latitude in *Festuca* [18], with Mesoamerican and northern Andean MCSA species showing lower ploidy levels (4×, and few 6×) and central and southern Andean species showing higher levels of ploidy (6×, 8×; except tetraploid *F. argentina*) (Table 2). Similar patterns of polyploid radiations have been reported for other angiosperms (e.g., C4 grasses, *Silene* L. [16,54]). This latitudinal change, also observed in species of *Festuca* from the Northern Hemisphere, has been related to the drastic effect of the Pleistocene glaciations and the successful postglacial colonization of high latitudinal and altitudinal territories by high polyploids [18]. For the MCSAI Glabricarpae, Asperifolia and *F. superba*, and MCSAII Erosiflorae, Ruprechtia and *F. argentina* lineages, the variations observed within clades in ploidy levels probably involved successive rounds of hybridizations and allopolyploidizations between these and/or other unstudied species that should be investigated through comparative genomic analyses.

### 3.2. Systematics of Broad-Leaved MCSA and Mallopetalon Loliinae Taxa

The morphological differences observed for the main diagnostic characters (Table 1) of MCSAII *F. argentina,* and MCSAI *F. superba* (Figure 2 and Figure 3b) with respect to the subgeneric or sectional *Festuca* ranks ascribed previously [31,46,48], motivated us to reclassify them (Table 1). *Festuca argentina*, traditionally classified within FL *F*. subgen. *Festuca* [46], shows a caespitose habit containing only intravaginal innovations, and plicate and junciform leaves with conduplicate vernation, which are different from those of all other broad-leaved taxa studied (Table 1, Figure 1 and Supplementary Figure S1). Dubcovsky [47] discussed the similarities between *F. argentina* and *F. fimbriata* (*F*. subgen. *Mallopetalon*), which share muticous or mucronulate lemma apices and hairy ovary tips (Table 1), and ciliate or fimbriated lodicles, 3-veined lower glumes and asymmetric and heterogeneous karyotypes. However, the same author indicated that *F. argentina* differed from *F. fimbriata* based on its intravaginal innovations, plicate leaves, smaller panicles and scabrid lemmas, and suggested a separate subgeneric classification for *F. argentina* [47]. *F. argentina* is nested within or sister to strongly supported Erosiflorae lineages in most nuclear and plastome phylogenies (Figure 2a,b,d and Figure 3b), supporting common ancestry with these taxa despite their disparate morphological traits (Table 1, Figure 1 and Supplementary Figure S1). This tetraploid species has a strongly asymmetric and heterogeneous karyotype, with two extremely discordant chromosomes sets [47], indicative of its allotetraploidy [51,52]. The species is, however, a low polyploid in its austral latitudinal distribution [32], which points to its relatively ancestral hybrid origin [25] and its plausible glacial survival and adaptation to the harsh climate conditions of the Patagonian steppe. One of its main distinguishing features, dioecy (Table 1), is shared with other species of its putative paternal Leucopoa ancestor, such as the North American *F. kingii* (Figure 2a,b and Figure 3b) and various Asian *F*. subgen. *Leucopoa* species [55,56]. As in the close genus *Poa* L., where hermaphroditism is the plesiomorphic state and dioecy has evolved in certain geographically distributed lineages in North and South America [57], the rare dioecy is restricted only to a few species of *Festuca* from Central and East Asia (e.g., *F. olgae* (Regel) Krivot., *F. sibirica* Hack. ex Boiss.) and their American descendants (*F. kingii*, *F. argentina*) ([55,56], this study). It is plausible to postulate that dioecy and chromosomal sex determination could have been maintained through allopolyploid speciation in *F. argentina*, as demonstrated in other angiosperms [58]. Based on the unique morphological characteristics displayed by *F. argentina* and its strong phylogenetic nesting within the Erosiflorae lineage of the MCSAII lineage, we propose to classify it within a new *Festuca* subgenus *Coironhuecu* Moreno-Aguilar, Arnelas & Catalán (see Taxonomic section below).

*Festuca superba* was misclassified into the artificially expanded *F*. subgen. *Erosiflorae* by Stančík and Renvoize [48]. However, this species differs morphologically from the species in this taxonomic rank as well as from the species of *F*. subgen. *Asperifolia* and *F*. subgen. *Subulatae* sect. *Glabricarpae* of the MCSAI clade where *F. superba* is evolutionarily positioned in all phylogenetic reconstructions (Table 1, Figure 1, Figure 2a–d and Figure 3b). The morphological features that characterize *F. superba*, such as the possession of broad and flat leaves with subconvolute vernation, entire and unawned lemmas, and a densely hairy ovary tip (Table 1 and Supplementary Figure S3), together with a shorter caryopsis hilum than the Erosiflorae taxa [32], approximate it to *F*. subgen. *Drymanthele* [35,55]. However, some private traits, such as the possession of a long hyaline ligule with an erose-dentate and ciliate apex (Table 1 and Figure 1), differentiate it from species of the sections described so far within this subgenus, namely European species of *F*. sect. *Phaeochloa* Griseb., Asian species of *F*. sect. *Muticae* S.L. Lu, and American and Australian species of *F*. sect. *Banksia* E.B. Alexeev [35,38,39,44,59]. Phylogenetically, some species of *F*. sect. *Banksia* were nested within either the FL clade (e.g., *F. purpurascens*, American I lineage) or within the BL clade (e.g., *F. muelleri* Vickery, Leucopoa-Amphigenes), while the studied species of *F*. sects. *Phaeochloa* (*F. altissima* All., *F. drymeja* Mert. & W.D.J. Koch, *F. lasto* Boiss., *F. donax* Lowe) and *Muticae* (*F. modesta* Nees) always nested within the BL clade [25]. *F. superba* is presumably an allooctoploid, based on its perfectly paired bivalents observed at meiosis [47]. Its high repeat content (Supplementary Table S1 and Figure 3a) and its recently evolved phylogenetic position in the nuclear and plastome trees (Figure 2a,b,d and Figure 3b) corroborate its plausible recent origin and lack of evolutionary time to purge its abundant repeatome [27]. Based on its particular morphological features, which approximate it to *F*. subgen. *Drymanthele* but not to currently described sections of this rank, and because of its strong phylogenetic nesting within the Glabricarpae–Asperifolia clades of the MCSAI lineage, we propose to tentatively classify it within *F*. subgen. *Drymanthele sensu lato* without a sectional assignment until other close broad-leaved Meso-South American taxa are also phylogenomically studied.

The systematics of Loliinae has undergone multiple classifications since the description of its main genus *Festuca* by Linné [23], resulting in the incorporation and segregation of new taxa to it. *Festuca* and fourteen close genera constitute the monophyletic subtribe Loliinae. Phylogenetic analysis has shown that fine-leaved *F*. subgen. *Festuca* species and some broad-leaved fescues (*F*. subgen. *Mallopetalon*, *F*. subgen. *Drymanthele* pro parte) plus ten annual genera (*Ctenopsis* De Not., *Dielsiochloa* Pilg., *Hellerochloa, Megalachne* Steud., *Micropyrum* (Gaudin) Link, *Narduroides* Rouy, *Podophorus* Phil., *Psilurus* Trin., *Vulpia* C.C. Gmel., *Wangenheimia* Moench) make up the FL clade, while taxa of eight broad-leaved *Festuca* subgenera (*F*. subgen. *Asperifolia*, *Drymanthele*, *Erosiflorae*, *Leucopoa, Schedonorus*, *Subulatae*, *Subuliflorae*, *Xanthochloa*) plus three annual or perennial genera (*Lolium* L., *Micropyropsis* Romero Zarco & Cabezudo, *Pseudobromus* K. Schum.) form the BL clade ([23,25,26,27], this study). The taxonomic distinction of these generic and infrageneric (*Festuca*) taxa is based on several diagnostic vegetative and reproductive morphoanatomical traits ([23], and references therein). Although none of the individual characteristics is absolute to identify a particular taxon, the combination of them has been used successfully to classify all these taxa in various floras and taxonomic treatments. In their systematic approach to subtribe Loliinae based on phylogenetic evidence, Catalán et al. [23] contemplated four potential scenarios for the classifications of the Loliinae (*Festuca sensu latissimo, sensu lato, sensu stricto, sensu strictissimo*). We propose to apply the *Festuca sensu lato* classification scenario, which is based on a systematic evolutionary criterion that is nomenclaturally conservative and maintains a paraphyletic *Festuca* (with subgenera and sections) and other traditionally recognized genera. Our current study has demonstrated the applicability of our systematic approach in the group of studied broad-leaved MCSA and Mallopetalon species, for which their phylogenetic resolution does not always coincide with their taxonomic classification as a consequence of the high reticulation of the Loliinae but has helped to disentangle their hybrid allopolyploid evolutionary history.

### 3.3. Description of Festuca subgen. Coironhuecu subgen. nov.

*Festuca* subgen. *Coironhuecu* Moreno-Aguilar, Arnelas & Catalán, *subgen. nov.*

Description: Perennial dioecious caespitose plant presenting intravaginal innovations, plicate and junciform leaves, short membranous ligule with a truncate and densely ciliate apex, erect narrowly lanceolate and contracted panicle, tri-nerved lower glume, muticous or mucronulate lemma apex and sparsely hispid ovary tip.

Typus: *Festuca argentina* (Speg.) Parodi, Physis (Buenos Aires) 11: 498. 1935. Basionym: *Poa argentina* Speg., Revista de la Facultad de Agronomía y Veterinaria 3 (30–31): 584–585. 1897. Ind. loc.: “Argentina: Hab. ad margim orientalem Lago Argentino, anno 1884”. Type specimen: Lago Argentino, 1884, *Sr. Tonini del Furia* s.n. (holotype, LP 001626; isotypes, BAA 2455, US 81670).

The subgenus is integrated only by *Festuca argentina* (Speg.) Parodi. It differs from the rest of the subgenera by the combination of its dioecy, caespitose habit, plicate leaves, tri-nerved lower glume, unawned lemma apex and sparsely hairy ovary tip. Etymology: *Coironhuecu* is based in the common Patagonian native name of *F. argentina* (Coirón huecú) due to its toxicity caused by its fungal endophytes.

## 4. Material and Methods

### 4.1. Morphological Study of Herbarium Festuca Specimens

Fifty herbarium specimens from AAU, BAA, MO, SI, US and UZ and 13 digital specimens (Supplementary File S1) from BAA, C, COL, IEB, K, LIL, LPB, MO and US were examined morphologically in search of the diagnostic characters provided by Alexeev and other authors to classify the Mesoamerican and South American *Festuca* species in the subgeneric and sectional taxa under study [30,31,37,38,39,41,42,44,46,48,50,60,61,62] and in other close morphological [32,33] and phylogenetic [24,25] taxa. We also evaluated 10 additional quantitative traits (culm height, ligule length, innovation leaf length, inflorescence length, inflorescence width, spikelet length, lower glume length, upper glume length, lemma length, awn length); however, none of them had a robust diagnostic value compared to the qualitative traits studied (Table 1). Ploidy levels were obtained from chromosome counts based on previous studies [18,22,23,24,25,26,27] and references therein. All *Festuca* species have a chromosome base number of x = 7; ploidy levels of the Meso and South American species studied (Table 2) fall within the expected range of known polyploid levels in the genus [18].

### 4.2. DNA Sampling of Festuca Species, Genome Sequencing, Data Assembling and Phylogenomic Analysis

Total DNA sampling was performed on representative species of all Mesoamerican and South American supraspecific *Festuca* ranks under study (Table 1 and Table 2). We also added a representative species of FL *F*. sect. *Eskia* (*F. gautieri*) to the analysis. DNA was isolated from herbarium specimens or silica gel dried samples using a modified CTAB protocol [63] with ∼20 mg of tissue. Genome skimming sequencing was performed from PCR-free libraries through the Illumina technology at the Spanish Centro Nacional de Análisis Genómicos (CNAG) and Macrogen, and the Illumina pair-end (PE) reads were processed following the procedures described in Moreno-Aguilar et al. [26].

Assembled plastomes for most of the newly sequenced samples were obtained with Novoplasty v. 2.7.1 [64] using the *F. pratensis* plastome (JX871941) as a reference and standardized parameters (k-mer: 29–39, insert size: ∼95–200 bp, genome range: 120,000–220,000 bp, PE reads: 101–150 bp). The plastomes of four samples with low number of PE reads (*F. asperella*, *F. breviglumis, F. valdesii, F. venezuelana,*) were assembled using a read-mapping strategy to, respectively, closely related *Festuca* plastomes using Geneious Prime 2022 (Table 2). The plastome sequences of another 14 representative Loliinae lineages were retrieved from previous studies [26,27].

The nuclear rDNA 45S region (transcribed cistron 5′-ETS-18S gene- ITS1-5.8S gene-ITS2-25S gene, plus intergenic sequence (IGS) region) of 27 of the 36 new Loliinae samples studied was extracted with the TAREAN tool of the Repeat Explorer2 (RE2) software [65,66] through the Galaxy platform on the ELIXIR public server (https://repeatexplorer-elixir.cerit-sc.cz accessed on 30 May 2022). Clustering was performed using default TAREAN tool settings (BLAST threshold of 90%, similarity across 55% of the read to identify reads to each cluster, minimum overlap = 55, cluster threshold = 0.01% input reads) and an input of 500,000 PE reads per sample. 45S rDNA sequences were found in the TAREAN tandem reports of each sample. The 45S region was divided into its 35S and IGS regions using the *Brachypodium distachyon* (L.) P. Beauv. 45S sequence as reference (Table 2). The nuclear rDNA 5S gene of most of the newly sequenced samples was also obtained with the RE2 TAREAN tool. The 45S sequences of nine species (*F. abyssinica* Hochst. ex A. Rich., *F. asperella*, *F. asplundii, F. capillifolia* Dufour ex Roem. & Schult., *F. fimbriata, F. kingii, F. pampeana* Speg., *F. quadridentata*, *F. venezuelana*) and the 5S sequences of two species (*F. asperella*, *F. venezuelana*) that could not be recovered by TAREAN were assembled employing a read-mapping strategy using, respectively, *F. triflora* J.F. Gmel. and *F. pratensis* as reference sequences in Geneious Prime 2022. Additional 35S and 5S sequences from other Loliinae lineages were retrieved from previous studies [26,27].

Entire plastomes and nuclear 35S, IGS and 5S sequences were aligned separately with MAFFT v. 7.031b [67]. TrimAl software v. 1.2rev59 [68] was used to remove low quality regions from each of the multiple sequence alignments (MSA) by imposing the -*automated*1 parameter. Maximum likelihood (ML) phylogenetic trees were reconstructed for each separated data set with Iqtree imposing the best-fit nucleotide substitution model, according to the Bayesian Information Criterion (BIC), and estimating 1000 ultrafast bootstrap replicates (BS) for the branch support of the best tree [69,70,71]. The Singular Value Decomposition quartets (SVDq) approach was implemented in Paup * [72], imposing nquartets = all seed = 2 nthreads = 4 bootstrap = 1000 options with a multispecies coalescent tree model and the quartet assembly algorithm QFM. Bootstrap support of the branches was shown in the tree obtained from SVD quartet analysis.

The composition and proportion of repetitive elements of the studied *Festuca* species were obtained from similarity graph-based clustering analysis of filtered PE reads using the Repeat Explorer pipeline of RE2 [66]. Previous studies have demonstrated that similarity-based clustering of low coverage genome sequencing reads, confidentially representing 0.50–0.01 of the total haploid genome coverage, is proportional to the genomic abundance and longitude of the corresponding repeat types in several angiosperm lineages and the Loliinae, and thus could be used to quantify them ([27], and references therein). The individual and comparative analyses of the studied samples was conducted following the procedures described in Moreno-Aguilar et al. [27]. Briefly, automated RE2 cluster annotation was used to quantify clusters and calculate the proportions of repetitive elements in each sample in the individual analysis (Supplementary Table S1). Comparative clustering analysis was performed for all the 36 samples studied in a single Galaxy run using the maximum number of randomly sampled PE reads that could be processed (~0.08–0.2 genome coverage for each species). Neighbor-Joining phylogenetic trees were computed for the top clusters selected in the comparative RE2 analysis with the NJ function of the ape package in R [73] using pairwise Euclidean genetic distances between the repeat contents of the species. Clusters with incomplete information (NA or zero values) for some samples were discarded from downstream analysis. A consensus network was constructed from all the repeat NJ trees with SplitsTree4 [74].

## Figures and Tables

**Table 1:** Morphological diagnostic traits used to classify species within *Festuca* subg. *Erosiflorae*, *F*. subg. *Drymanthele* sect. *Ruprechtia*, *F*. subg. *Subulatae* sect. *Glabricarpae*, *F*. subg. *Asperifolia* and *F.* subgen. *Mallopetalon sensu* Alexeev and other authors, plus the newly described *F*. subgen. *Coironhuecu subgen. nov*. (*F. argentina*) and *F*. subgen. *Drymanthele sensu lato* (*F. superba*) analyzed in this study. The type of species of each subgeneric or sectional taxa are highlighted in bold. The asterisks indicate the species used in the phylogenomic analysis.

*Festuca* Subgenera, Sections, Species/Morphological Diagnostic Traits	Subgen. *Erosiflorae* *sensu* Alexeev: F. dichoclada * F. horridula * F. quadridentata* sensu Stančík & Peterson: *F. carrascana* *F. chuquisacae* *F. urubambana*	Subgen. *Drymanthele* Sect. *Ruprechtia* *sensu* Alexeev: F. amplissima* F. jaliscana *sensu* Gonzalez-Ledesma et al.: F. valdesii *	Subgen. *Subulatae* Sect. *Glabricarpae sensu* Alexeev: F. breviglumis* F. chiriquensis* F. dentiflora *F. steinbachii* F. caldasii* F. woodii This Study: F. venezuelana *	Subgen. *Asperifolia* *sensu* Alexeev: *F. lugens* F. asperella * F. tancitaroensis	Subgen. *Mallopetalon* *sensu* Alexeev: F. fimbriata *	Subgen. *Coironhuecu* *subgen. nov.* *This Study:* F. argentina *	Subgen. *Drymanthele* *sensu lato* (Without Sectional Assignation) This Study: F. superba *
Reproduction	Monoecious	Monoecious	Monoecious	Monoecious	Monoecious	Dioecious	Monoecious
Habit	Largely tussocked or rhizomatous or mixed	Rhizomatous or caespitose	Rhizomatous or loosely tufted	Densely tussocked or rhizomatous	Rhizomatose	Caespitose	Laxely caespitose to rhizomatose
Innovations	Extravaginal or intravaginal	Extravaginal or/and intravaginal	Extravaginal	Extravaginal or intravaginal	Extravaginal	Intravaginal	Mixed
Ligule	Membranaceous, apex acute, erose or lacerate, 5.5–21 mm long	Non-membranaceous, apex truncate shortly ciliate, or short membranaceous, apex truncate and ciliate; 0.1–0.5 (1) mm long	Membranceous or hyaline, apex truncate or rounded, lacerate or dentate; or shortly ciliate; 0.3–4 mm long	Membranaceous, apex truncate or slightly rounded and lacerate or dentate, 1.4–8 mm long	Membranaceous, apex truncate, erose and ciliate, 0.5–1.5 mm long	Membranaceous, apex truncate and densely ciliate, 0.4–1.5 mm long	Hyaline, apex truncate, erose and dentate, 2.7–5.5 mm long
Leaf blade	Flat, involute in the middle and subconvolute at the apex	Flat, involute in the middle and subconvolute at the apex	Flat, involute in the middle and subconvolute at the apex	Flat, involute in the middle and subconvolute at the apex	Largely flat	Plicate, junciform	Largely flat, subconvolute
Inflorescence	Erect	Erect	Nutant or erect with nutant branches	Erect or scarcely nutant	Erect, lax	Erect, contracted	Erect, branches flexuous
Lemma apex	Dentate or entire, unawned	Entire, unawned	Entire or bifid, awned	Bifid, shortly awned or unawned	Entire, scariose, rolled and fimbriate, unawned, muticous	Entire, unawned, muticous or mucronulate	Entire, unawned, muticous
Ovary tip	Glabrescent	Glabrous or hispid	Glabrous or sparsely hispid	Glabrous or hispid	Densely hairy	Sparsely hispid	Densely hairy

**Table 2:** Taxa included in the phylogenomic analysis of Mesoamerican and South American polyploid broad-leaved *Festuca* grasses. Taxon name and authorship, Loliinae phylogenetic lineage, ploidy level, locality of collection and voucher information, number of genomic Illumina pair-end read sequences, and GenBank accession codes for nuclear rDNA 35S cistron, (45S) IGS and 5S gene regions, and plastome sequences are given for each sample. Values in bold correspond to new data generated in this study. Ploidy levels are based on chromosome counts from previous studies (all *Festuca* species show the same chromosome base number of x = 7) [18,22,23,24,25,26,27] and references therein. Question mark: unknown ploidy level.

Taxon	Phylogenetic Lineage	Ploidy	Locality/Voucher	Illumina PE Reads (Millions)	GenBank Accession No.			
					35S	IGS	5S	Plastome
**Broad-Leaved (BL) Loliinae**								
*Festuca asperella* E.B. Alexeev	Asperifolia (MCSAI)	?	Mexico: Mexico DF; MO 2744225	**5683**	**OP120918**	**OP158136**	**OP142677**	**SAMN30029288**
*Festuca breviglumis* Swallen	Glabicarpae	?	Mexico: Mexico DF; P, M, Peterson 21366; US s.n	**12,181**	**OP120919**	**OP158139**	**OP142678**	**SAMN30029289**
*Festuca caldasii* (Kunth) Kunth	Glabicarpae (MCSAI)	4×	Ecuador: Catamayo, Chinchas-Tambara; HUTPL14055	9863	MT145280	**OP158140**	ON248977	SAMN14647047
*Festuca chiriquensis* Swallen	Glabicarpae (MCSAI)	4×	Costa Rica: Cartago, Cantón Turrialba; MO 5175763	**8653**	**OP120920**	**OP158143**	**OP142679**	**SAMN30029290**
*Festuca superba* Parodi ex Türpe	Drymanthele s. l. (MCSAI)	8×	Argentina: Jujuy, Yala, Laguna Rodeo; PC 356.08 UZ	12,193	MT145305	**OP158163**	ON248977	SAMN14647072
*Festuca venezuelana* Stančík	Glabicarpae (MCSAI)	6×	Venezuela: Tachira, La Grita; AAU-4262	**7957**	**OP120926**	**OP158166**	**OP142686**	**SAMN30029296**
*Festuca dichoclada* Pilg.	Erosiflorae (MCSAII)	?	Peru: Cuzco, Quispicanchi; P, M, Peterson 20603; US s.n.	**12,466**	**OP120921**	**OP158144**	**OP142680**	**SAMN30029291**
*Festuca horridula* Pilg.	Erosiflorae (MCSAII)	?	Peru: Junín, Yauli; Tovar, O, and H, Soplín 6607	**32,417**	**OP120923**	**OP158150**	**OP142682**	**SAMN30029293**
*Festuca quadridentata* Kunth	Erosiflorae (MCSAII)	?	Ecuador: Chimborazo, Alao; US 1911313	15,091	MT145303	**OP158160**	**OP142684**	SAMN14647070
*Festuca amplissima* Rupr.	Ruprechtia (MCSAII)	6×	Mexico: Nuevo Leon; Peterson 21097, US s.n.	12,058	MT145278	**OP158134**	ON248975	SAMN14647045
*Festuca valdesii* Gonz.-Led. & S.D. Koch	Ruprechtia (MCSAII)	?	Mexico: Coahuila; P, M, Peterson 21456; US s.n.	**10,937**	**OP120925**	**OP158165**	**OP142685**	**SAMN30029295**
*Festuca argentina* (Speg.) Parodi	Coironhuecu (MCSAII)	4×	Argentina: Rio Negro, Bariloche; PC, 0210	**22,928**	**OP120917**	**OP158135**	**OP142676**	**SAMN30029287**
*Festuca kingii* (S. Watson) Cassidy	Leucopoa	8×	USA: California: San Bernardino Mnts, Leg: Quibell 149; LE	**12,397**	**OP120924**	**OP158151**	**OP142683**	**SAMN30029294**
*Festuca spectabilis* Bertol.	Leucopoa	6×	Bosnia-Hercegovina: Troglav, Sajkovacko zdrlo, UZ	12,960	MT145304	**OP158162**	ON249004	SAMN14647071
*Festuca africana* (Hack.) Clayton	Tropical and South African	10×	Uganda: Gahinga; Namaganda 190Vg; MHU1603	13,549	MT145277	**OP158133**	ON248974	SAMN14647044
*Festuca mekiste* Clayton	Tropical and South African	?	Kenya: Mt, Elgon National Park, Kambi Mtamaiwa; Carvalho 4521	16,245	ON243855	**OP158153**	ON248992	SAMN27777779
*Festuca durandoi* Clauson	Subbulbosae	2×	Portugal: Serra Arga Alto do Espinheiro; UZ s.n.	12,688	MT145283	**OP158145**	ON248980	SAMN14647050
*Festuca paniculata* (L.) Schinz & Thell	Subbulbosae	2×	Spain: Caceres, Puerto de los Castaños; UZ 40.07	35,808	MT145297	**OP158157**	ON248996	SAMN14647064
*Festuca triflora* J.F. Gmel.	Lojaconoa	2×	Morocco: Rif Mountains, Bab Barret-Ketama; PC 39.17 UZ	24,472	MT145306	**OP158164**	ON249006	SAMN14647073
*Festuca lasto* Boiss.	Drymanthele (Phaeochloa)	2×	Spain: Cadiz, Los Alcornocales; UZ 29.08	21,581	MT145291	**OP158152**	ON248989	SAMN14647058
*Festuca pratensis* Huds.	Schedonorus	2×	UK: England; USDA PI 283306	12,189	MT145301	**OP158158**	ON248998	SAMN14647066
*Festuca arundinacea* subsp. *atlantigena* (St.-Yves) Auquier	Schedonorus	8×	Morocco: Atlas mountains; ABY BN 807	15,091	ON243851	**OP158138**	ON248990	SAMN27777775
*Festuca molokaiensis* Soreng, P.M. Peterson & Catalán	Subulatae-Hawaiian	?	USA: Hawaii: Molokai, BISH 728771	12,188	MT145294	**OP158154**	ON248993	SAMN14647061
**Fine-leaved (FL) Loliinae**								
*Festuca fimbriata* Nees	American II	6×	Argentina: Misiones, Dpto, Apóstoles; UZ 498.08	15,741	MT145286	**OP158146**	ON248983	SAMN14647053
*Festuca asplundii* E.B. Alexeev	American II	6×	Ecuador: Loja, Saraguro; HUTPL14046	25,088	MT145279	**OP158137**	ON248976	SAMN14647046
*Festuca procera* Kunth	American II	?	Ecuador: Riobamba, Chimborazo; HUTPL14079	40,669	MT145299	**OP158159**	ON248999	SAMN14647067
*Festuca chimborazensis* E.B. Alexeev	American I	6×	Ecuador: Riobamba, Chimborazo; HUTPL14066	10,913	MT145282	**OP158142**	ON248979	SAMN14647049
*Festuca holubii* Stančík	American I	?	Ecuador: Saraguro, to Cerro de Arcos; HUTPL14071	10,264	MT145289	**OP158149**	ON248988	SAMN14647056
*Festuca pampeana* Speg.	American Pampas	8×	Argentina: Buenos Aires, Sierra de la Ventana; PC 428.08	14,862	MT145296	**OP158156**	ON248995	SAMN14647063
*Festuca gracillima* Hook. f.	American–Neozeylandic I	6×	Argentina: Tierra de Fuego, E. San Pablo; UZ 482.08	13,888	MT145288	**OP158148**	ON248986	SAMN14647055
*Festuca abyssinica* Hochst. ex A. Rich.	Afroalpine	4×	Tanzania: Kilimanjaro; Afroalp O-DP-42737	12,041	MT145276	**OP158132**	ON248973	SAMN14647043
*Festuca rubra* L.	Aulaxyper	6×	Argentina: Tierra de Fuego, Cabo Annicolta; UZ 03.09	25,260	ON243856	**OP158161**	ON249002	SAMN27777780
*Festuca ovina* L.	Festuca	2×	Germany: Thüringen; Müller 10789	11,364	MT145295	**OP158155**	ON248994	SAMN14647062
*Festuca capillifolia* Dufour ex Roem. & Schult.	Exaratae	2×	Morocco: Middle Atlas, Ifrane National Park; PC 77.17	13,430	MT145281	**OP158141**	ON248978	SAMN14647048
*Vulpia ciliata* Dumort.	Psilurus–Vulpia	4×	Spain: Toledo, Mar de Ontígola; UZ 109.07	11,801	MT145309	**OP158167**	ON249009	SAMN14647076
*Festuca gautieri* (Hack.) K. Richt.	Eskia	2×	Spain: Granada, Huéscar; UZ 232.07	**13,941**	**OP120922**	**OP158147**	**OP142681**	**SAMN30029292**
**Outgroups**								
*Brachypodium distachyon* (L.) P. Beauv.	---	2×	Spain: Caceres; UZ 28.07	---	Phytozome Bd21 v.3.1	---	---	NC_011032, 1
*Oryza sativa* L.	---	2×	China: National Rice Research Center, cv	---	AP008215	---	---	AY522331, 1

## Data Availability

Input and output data, and Supplementary Information are available at Github (https://github.com/Bioflora/Erosiflorae (accessed on 29 August 2022)).

## Supplementary Materials

The following supporting information can be downloaded at: https://www.mdpi.com/article/10.3390/plants11172303/s1, Figure S1: Anatomical leaf blade section of representative species of Mesoamerican and South-American broad-leaved *Festuca* taxa analyzed morphologically in this study. *F*. subgen. *Subulatae* sect. *Glabricarpae*: *F. venezuelana* (**a**); *F*. subgen. *Drymanthele s. l*.: *F. superba* (**b**); *F*. subgen. *Subulatae* sect. *Glabricarpae*: *F. breviglumis* (**c**); *F*. subgen. *Asperifolia*: *F. asperella* (**d**); *F*. subgen. *Erosiflorae*: *F. quadridentata* (**e**), *F. dichloclada* **(f**); *F*. subgen. *Drymanthele* sect. *Ruprechtia*: *F. amplissima* (**g**); *F.* subgen. *Coironhuecu* (*subgen. nov*.): *F. argentina* (**h**); *F*. subgen. *Mallopetalon*: *F. fimbriata* (**i**). Drawings by José Alfredo Hidalgo-Salazar (**a**–**h**) and María Fernanda Moreno-Aguilar (**i**). [**a**: modified from Stančik & Peterson [31]; **b**: modified from Türpe [49]; c: Peterson PM. & Rosales O. 16117, US- 3524155; **d**: modified from Alexeev [38]; **e**: modified from St. Yves [46]; **f**: Smith et al. 10782, AAU; **g**: modified from Stančik & Peterson [31]; **h**: modified from Catalán & Muller [32]; **i**: Kostling M. 44, UZ 498.08]; Figure S2: Loliinae coalescent species trees computed through Singular Value Decomposition quartets (SVDq) analysis showing bootstrap support values on branches. (**a**) nuclear rDNA 35S tree; (**b**) nuclear rDNA (45S) IGS tree; (**c**) nuclear rDNA 5S tree; (**d**) plastome tree. *Oryza sativa* and *Brachypodium distachyon* outgroups were used to root some trees. Color codes of Loliinae lineages correspond to those indicated in the chart in Figure S2a. Scale bar: number of mutations per site; Figure S3: Morphological diagnostic traits mapped onto a Maximum Likelihood IGS cladogram tree of the Mesoamerican and South-American broad-leaved Festuca taxa studied and other representative species of the broad-leaved (BL) and fine-leaved (FL) Loliinae lineages. Traits codes: 1. Reproduction: monoecious (0), dioecious (1); 2. Habit: rhizomatous or caespitose or mixed (0), rhizomatose (1), caespitose (2); 3. Innovations: Extravaginal or intravaginal (0), intravaginal (2), extravaginal or/and intravaginal (3); 4. Ligule: membranaceous, apex acute, erose or lacerate, long (0), non- membranaceous, apex truncate shortly ciliate, or short membranaceous, apex truncate and ciliate, short (1), membranceous or hyaline, apex truncate or rounded, lacerate or dentate, or shortly ciliate, medium (2); membranaceous, apex truncate or slightly rounded and lacerate or dentate, medium-long (3); membranaceous, apex truncate, erose and ciliate, short (4); membranaceous, apex truncate and densely ciliate, short (5); 5. Leaf-blade: Flat, involute in the middle and subconvolute at the apex (0), largely flat (1), plicate, junciform (2), largely flat, subconvolute (3); 6. Inflorescence: erect (0), nutant or erect with nutant branches (1), erect or scarcely nutant (2), erect, laxe (3), erect, contracted (4), erect, branches flexuous (5); 7. Lemma apex: dentate or entire, unawned (0), entire, unawned (1), entire or bifid, awned (2), bifid, shortly awned or unawned (3), entire, scariose, rolled and fimbriate, unawned, muticous (4), entire, unawned, muticous or mucronulate (5), entire, unawned, muticous (6); 8. Ovary tip: glabrescent (0), glabrous or hispid (1), densely hairy (2), sparsely hispid (3); File S1: List of 65 specimens examined taxonomically of the species under study [*Festuca* subgen. *Erosiflorae*, *F*. subgen. *Drymanthele* sect. *Ruprechtia*, *F*. subgen. *Subulatae* sect. *Glabricarpae*, *F*. subgen. *Asperifolia* and *F*. subgen. *Mallopetalon* sensu Alexeev, plus the newly described *F*. subgen. *Coironhuecu subgen. nov*. (*F. argentina*) and *F.* subgen. *Drymanthele sensu lato* (*F. superba*)], ranked in alphabetical order; Table S1: Genome proportion of repeats estimated by Repeat Explorer2 for individual Loliinae samples (estimated percentages per holoploid genome, 1C). Values in bold correspond to new data generated in this study.
