# Supplementary material for: Phylogenomics and Systematics of Overlooked Mesoamerican and South American Polyploid Broad-Leaved Festuca Grasses Differentiate F. sects. Glabricarpae and Ruprechtia and F. subgen. Asperifolia, Erosiflorae, Mallopetalon and Coironhuecu (subgen. nov.)
Source: Plants (Basel). 2022 Sep 2;11(17):2303. doi: 10.3390/plants11172303 (PMC9460391; doi:10.3390/plants11172303)
Supplement: Supplementary file 1 [file plants-11-02303-s001.zip › supplementary Figures.pdf]

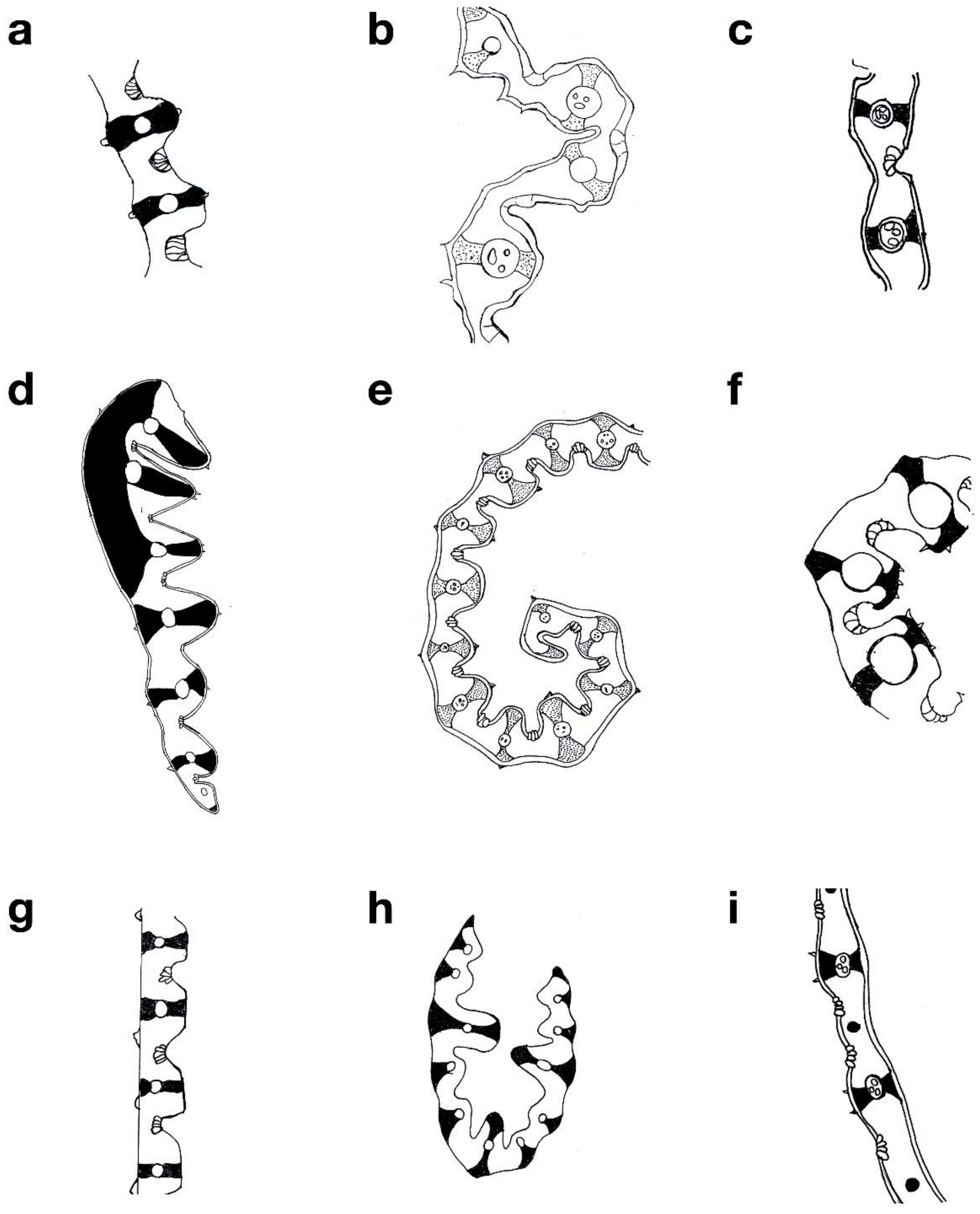

Figure S1. Anatomical leaf blade section of representative species of Mesoamerican and South-American broad-leaved *Festuca* taxa analyzed morphologically in this study. *F.* subgen. *Subulatae* sect. *Glabricarpae*: *F. venezuelana* (**a**); *F.* subgen. *Drymanthele s. l.*: *F. superba* (**b**); *F.* subgen. *Subulatae* sect. *Glabricarpae*: *F. breviglumis* (**c**); *F.* subgen. *Asperifolia*: *F. asperella* (**d**); *F.* subgen. *Erosiflorae*: *F. quadridentata* (**e**), *F. dichloclada* (**f**); *F.* subgen. *Drymanthele* sect. *Ruprechtia*: *F. amplissima* (**g**); *F.* subgen. *Coironhuecu* (*subgen. nov.*): *F. argentina* (**h**); *F.* subgen. *Mallopetalon*: *F. fimbriata* (**i**). Drawings by José Alfredo Hidalgo-Salazar (**a-h**) and María Fernanda Moreno-Aguilar (**i**). [**a**: modified from Stančik & Peterson [31]; **b**: modified from Türpe [74]; c: Peterson PM. & Rosales O. 16117, US- 3524155; **d**: modified from Alexeev [38]; **e**: modified from St. Yves [46] ; **f**: Smith et al. 10782, AAU; **g**: modified from Stančik & Peterson [31] ; **h**: modified from Catalán & Muller [32]; **i**: Kostling M. 44, UZ 498.08].

a

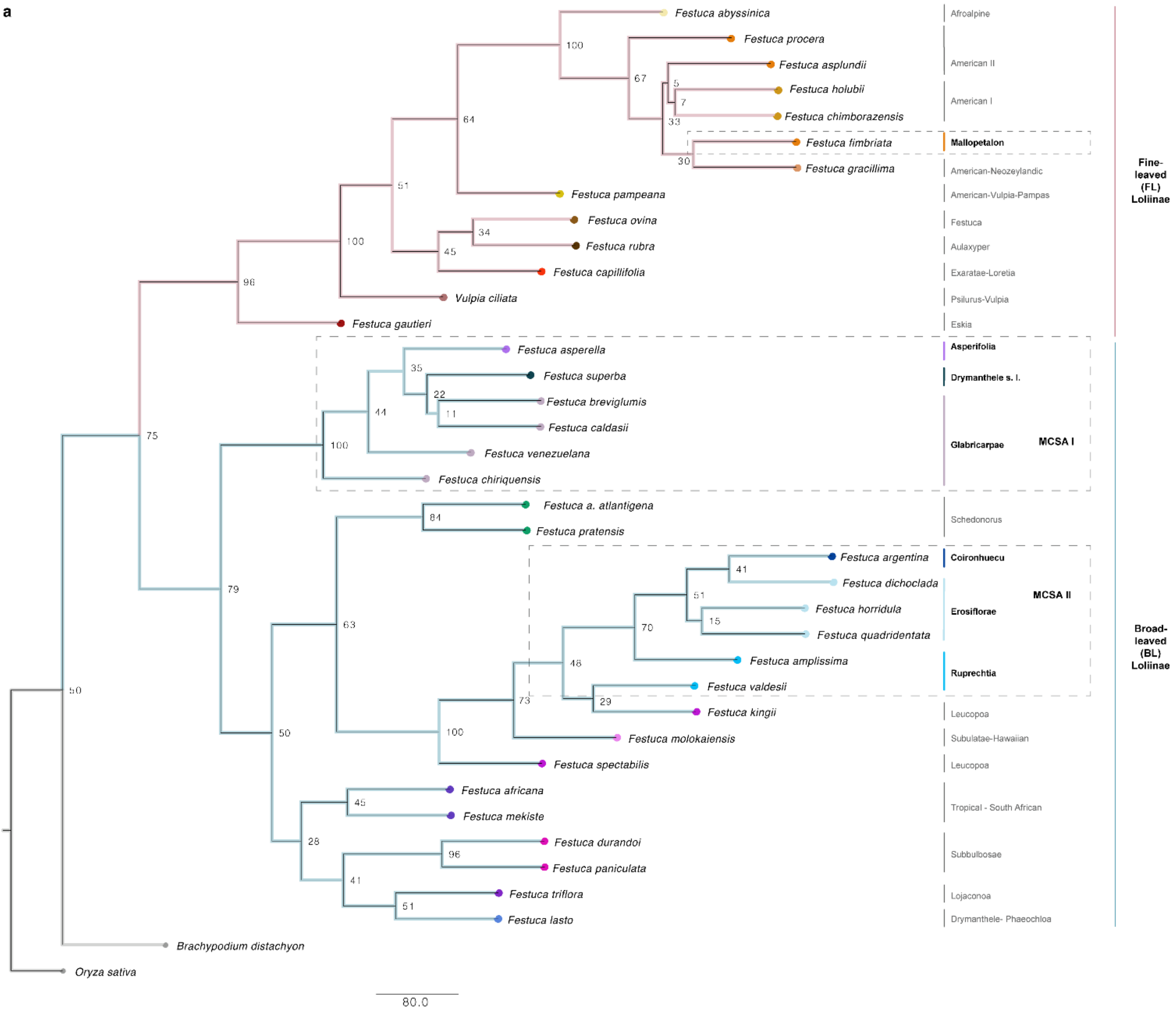

Festuca abyssinica
Festuca procera
Festuca asplundii
Festuca holubii
Festuca chimborazensis
Festuca fimbriata
Festuca gracillima
Festuca pampeana
Festuca ovina
Festuca rubra
Festuca capillifolia
Vulpia ciliata
Festuca gautieri
Festuca asperella
Festuca superba
Festuca breviglumis
Festuca caldasii
Festuca venezuelana
Festuca chiriquensis
Festuca a. atlantigena
Festuca pratensis
Festuca argentina
Festuca dichoclada
Festuca horridula
Festuca quadridentata
Festuca amplissima
Festuca valdesii
Festuca kingii
Festuca molokaiensis
Festuca spectabilis
Festuca africana
Festuca mekiste
Festuca durandoi
Festuca paniculata
Festuca triflora
Festuca lasto
Brachypodium distachyon
Oryza sativa
Afroalpine
American II
American I
Mallopetalon
American-Neozeylandic
American-Vulpia-Pampas
Festuca
Aulaxyper
Exaratae-Loretia
Psilurus-Vulpia
Eskia
Asperifolia
Drymanthele s. l.
Glabricarpae
MCSA I
Schedonorus
Coironhuecu
Erosiflorae
MCSA II
Ruprechtia
Leucopoa
Subulatae-Hawaiian
Leucopoa
Tropical - South African
Subbulbosae
Lojaconoa
Drymanthele- Phaeochloa
Fine-leaved (FL) Loliinae
Broad-leaved (BL) Loliinae
80.0

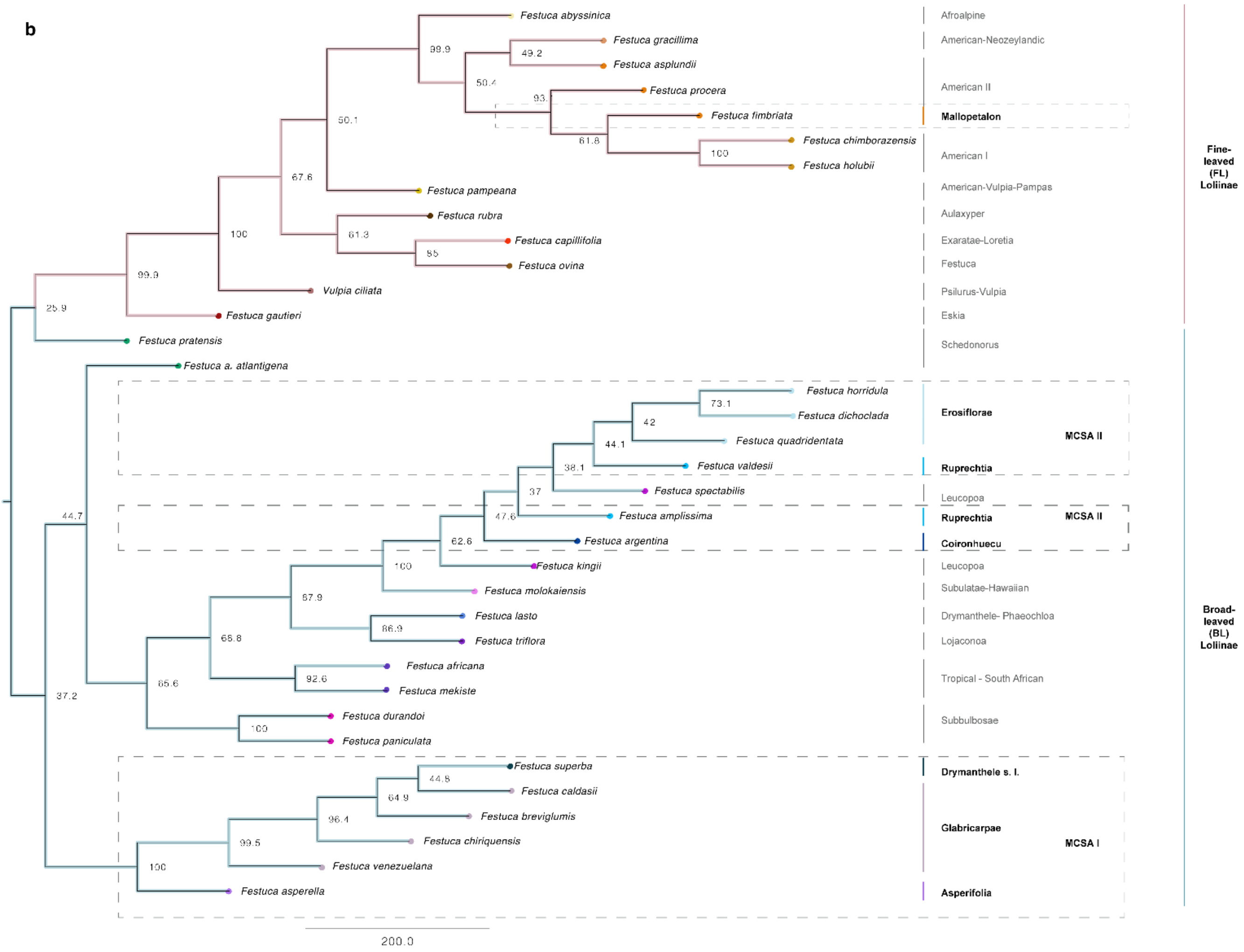
b
Festuca abyssinica
Festuca gracillima
Festuca asplundii
Festuca procera
Festuca fimbriata
Festuca chimborazensis
Festuca holubii
Festuca pampeana
Festuca rubra
Festuca capillifolia
Festuca ovina
Vulpia ciliata
Festuca gautieri
Festuca pratensis
Festuca a. atlantigena
Festuca horridula
Festuca dichoclada
Festuca quadridentata
Festuca valdesii
Festuca spectabilis
Festuca amplissima
Festuca argentina
Festuca kingii
Festuca molokaiensis
Festuca lasto
Festuca triflora
Festuca africana
Festuca mekiste
Festuca durandoi
Festuca paniculata
Festuca superba
Festuca caldasii
Festuca breviglumis
Festuca chiriquensis
Festuca venezuelana
Festuca asperella
Afroalpine
American-Neozeylandic
American II
Mallopetalon
American I
American-Vulpia-Pampas
Aulaxyper
Exaratae-Loretia
Festuca
Psilurus-Vulpia
Eskia
Schedonorus
Erosiflorae
Ruprechtia
Leucopoa
Ruprechtia
Coironhuecu
Leucopoa
Subulatae-Hawaiian
Drymanthele- Phaeochloa
Lojaconoa
Tropical - South African
Subbulbosae
Drymanthele s. l.
Glabricarpae
Asperifolia
MCSA II
MCSA II
MCSA I
Fine-leaved (FL) Loliinae
Broad-leaved (BL) Loliinae
200.0

c

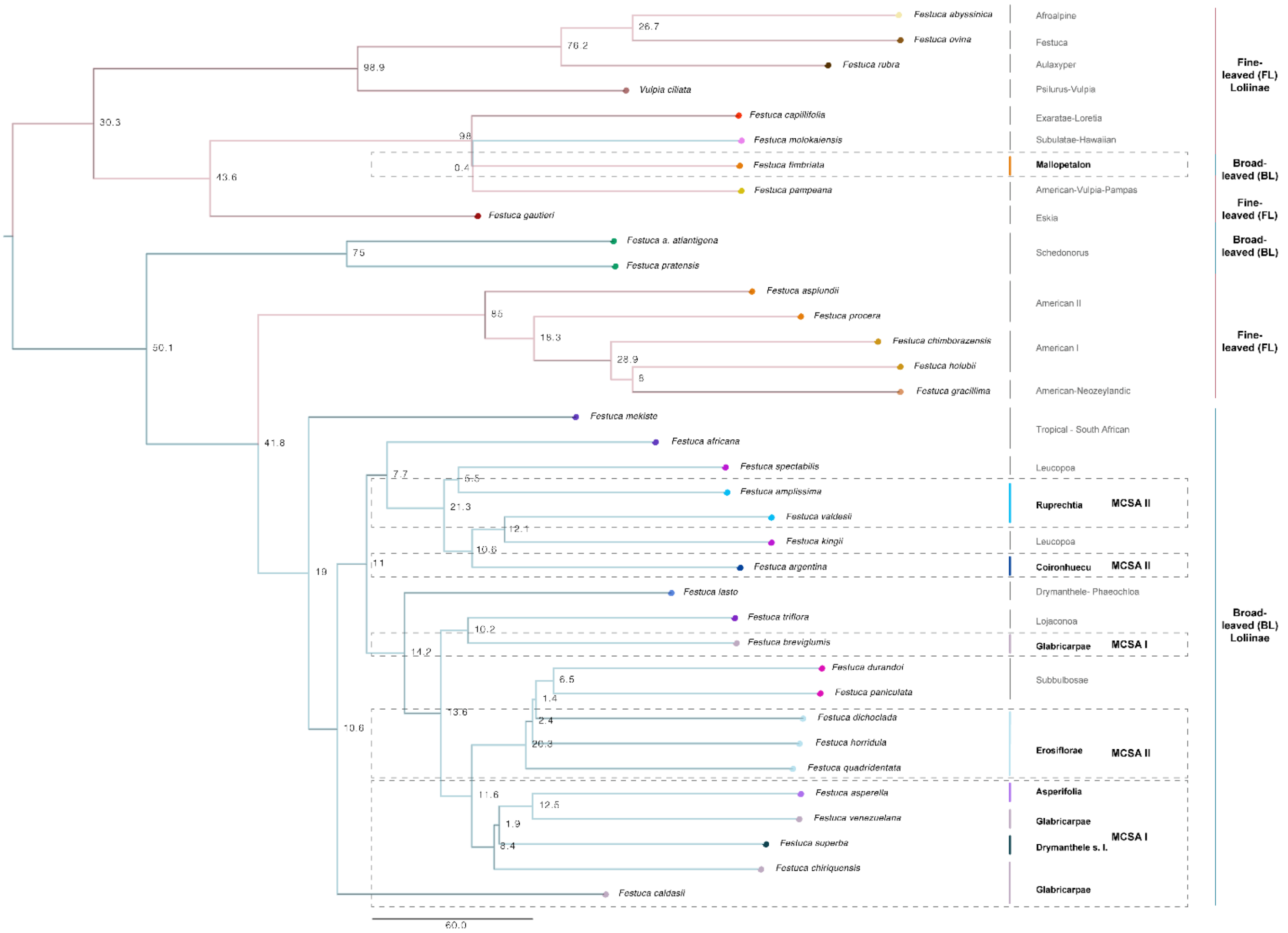
Festuca abyssinica
Festuca ovina
Festuca rubra
Vulpia ciliata
Festuca capillifolia
Festuca molokaiensis
Festuca fimbriata
Festuca pampeana
Festuca gautieri
Festuca a. atlantigena
Festuca pratensis
Festuca asplundii
Festuca procera
Festuca chimborazensis
Festuca holubii
Festuca gracillima
Festuca mekiste
Festuca africana
Festuca spectabilis
Festuca amplissima
Festuca valdesii
Festuca kingii
Festuca argentina
Festuca lasto
Festuca triflora
Festuca breviglumis
Festuca durandoi
Festuca paniculata
Festuca dichoclada
Festuca horridula
Festuca quadridentata
Festuca asperella
Festuca venezuelana
Festuca superba
Festuca chiriquensis
Festuca caldasii
Afroalpine
Festuca
Aulaxyper
Psilurus-Vulpia
Exaratae-Loretia
Subulatae-Hawaiian
Mallopetalon
American-Vulpia-Pampas
Eskia
Schedonorus
American II
American I
American-Neozeylandic
Tropical - South African
Leucopoa
Ruprechtia
MCSA II
Leucopoa
Coironhuecu
MCSA II
Drymanthele- Phaeochloa
Lojaconoa
Glabricarpae
MCSA I
Subbulbosae
Erosiflorae
MCSA II
Asperifolia
Glabricarpae
Drymanthele s. l.
MCSA I
Glabricarpae
Fine-leaved (FL) Loliinae
Broad-leaved (BL)
Fine-leaved (FL)
Broad-leaved (BL)
Fine-leaved (FL)
Broad-leaved (BL) Loliinae
60.0

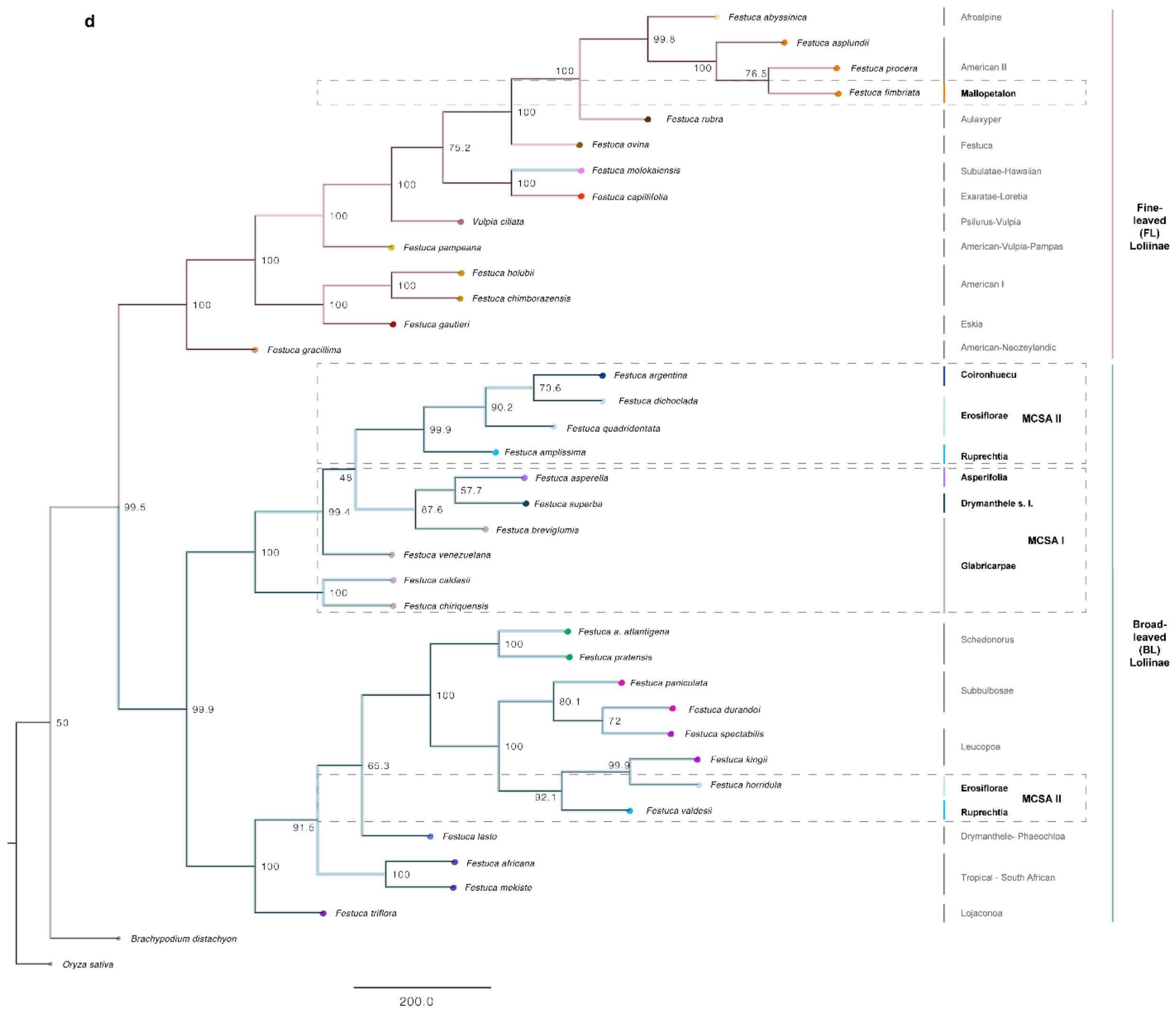

Figure S2. Loliinae coalescent species trees computed through Singular Value Decomposition quartets (SVDq) analysis showing bootstrap support values on branches. (**a**) nuclear rDNA 35S tree; (**b**) nuclear rDNA (45S) IGS tree; (**c**) nuclear rDNA 5S tree; (**d**) plastome tree. *Oryza sativa* and *Brachypodium distachyon* outgroups were used to root some trees. Color codes of Loliinae lineages correspond to those indicated in the chart in Figure 2a. Scale bar: number of mutations per site.

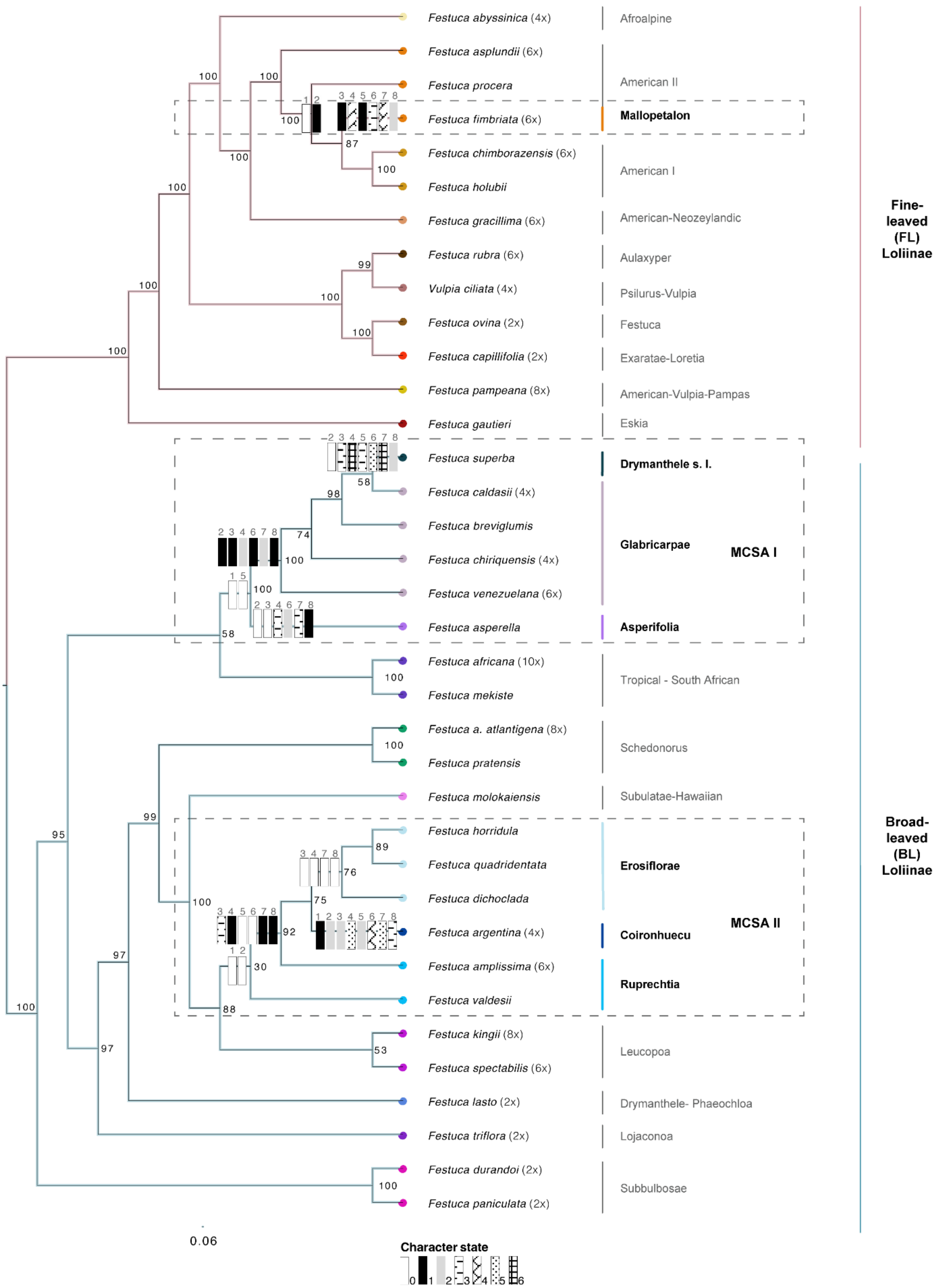
Festuca abyssinica (4x)
Festuca asplundii (6x)
Festuca procera
Festuca fimbriata (6x)
Festuca chimborazensis (6x)
Festuca holubii
Festuca gracillima (6x)
Festuca rubra (6x)
Vulpia ciliata (4x)
Festuca ovina (2x)
Festuca capillifolia (2x)
Festuca pampeana (8x)
Festuca gautieri
Festuca superba
Festuca caldasii (4x)
Festuca breviglumis
Festuca chiriquensis (4x)
Festuca venezuelana (6x)
Festuca asperella
Festuca africana (10x)
Festuca mekiste
Festuca a. atlantigena (8x)
Festuca pratensis
Festuca molokaiensis
Festuca horridula
Festuca quadridentata
Festuca dichoclada
Festuca argentina (4x)
Festuca amplissima (6x)
Festuca valdesii
Festuca kingii (8x)
Festuca spectabilis (6x)
Festuca lasto (2x)
Festuca triflora (2x)
Festuca durandoi (2x)
Festuca paniculata (2x)
Afroalpine
American II
Mallopetalon
American I
American-Neozeylandic
Aulaxyper
Psilurus-Vulpia
Festuca
Exaratae-Loretia
American-Vulpia-Pampas
Eskia
Drymanthele s. l.
Glabricarpae
MCSA I
Asperifolia
Tropical - South African
Schedonorus
Subulatae-Hawaiian
Erosiflorae
Coironhuecu
MCSA II
Ruprechtia
Leucopoa
Drymanthele- Phaeochloa
Lojaconoa
Subbulbosae
Fine-leaved (FL) Loliinae
Broad-leaved (BL) Loliinae
0.06
Character state
0 1 2 3 4 5 6

Figure S3. Morphological diagnostic traits mapped onto a Maximum Likelihood IGS cladogram tree of the Mesoamerican and South-American broad-leaved *Festuca* taxa studied and other representative species of the broad-leaved (BL) and fine-leaved (FL) Loliinae lineages. Traits codes: 1. Reproduction: monoecious (0), dioecious (1); 2. Habit: rhizomatous or caespitose or mixed (0), rhizomatose (1), caespitose (2); 3. Innovations: Extravaginal or intravaginal (0), intravaginal (2), extravaginal or/and intravaginal (3); 4. Ligule: membranaceous, apex acute, erose or lacerate, long (0), non- membranaceous, apex truncate shortly ciliate, or short membranaceous, apex truncate and ciliate, short (1), membranceous or hyaline, apex truncate or rounded, lacerate or dentate, or shortly ciliate, medium (2); membranaceous, apex truncate or slightly rounded and lacerate or dentate, medium-long (3); membranaceous, apex truncate, erose and ciliate, short (4); membranaceous, apex truncate and densely ciliate, short (5); 5. Leaf-blade: Flat, involute in the middle and subconvolute at the apex (0), largely flat (1), plicate, junciform (2), largely flat, subconvolute (3); 6. Inflorescence: erect (0), nutant or erect with nutant branches (1), erect or scarcely nutant (2), erect, laxe (3), erect, contracted (4), erect, branches flexuous (5); 7. Lemma apex: dentate or entire, unawned (0), entire, unawned (1), entire or bifid, awned (2), bifid, shortly awned or unawned (3), entire, scariose, rolled and fimbriate, unawned, muticous (4), entire, unawned, muticous or mucronulate (5), entire, unawned, muticous (6); 8. Ovary tip: glabrescent (0), glabrous or hispid (1), densely hairy (2), sparsely hispid (3).
