# Supplementary material for: Phylogenomics and Systematics of Overlooked Mesoamerican and South American Polyploid Broad-Leaved Festuca Grasses Differentiate F. sects. Glabricarpae and Ruprechtia and F. subgen. Asperifolia, Erosiflorae, Mallopetalon and Coironhuecu (subgen. nov.)
Source: Plants (Basel). 2022 Sep 2;11(17):2303. doi: 10.3390/plants11172303 (PMC9460391; doi:10.3390/plants11172303)
Supplement: Supplementary file 1 [file plants-11-02303-s001.zip › Supplementary File S1 & Table S1.pdf]

**Supplementary File S1**. List of 65 specimens examined taxonomically of the species under study [*Festuca* subgen. *Erosiflorae*, *F.* subgen. *Drymanthele* sect. *Ruprechtia*, *F.* subgen. *Subulatae* sect. *Glabricarpae*, *F.* subgen. *Asperifolia* and *F.* subgen. *Mallopetalon* sensu Alexeev, plus the newly described *F.* subgen. *Coironhuecu subgen. nov.* (*F. argentina*) and *F.* subgen. *Drymanthele sensu lato* (*F. superba*)], ranked in alphabetical order.

***Festuca amplissima* Rupr.** MEXICO. México: 3.1 mi SE Plan de Vegas [Vigas], 11 October 2001, Peterson P.M. 16150 & Herrera-Arrieda Y. (US). ***Festuca argentina* (Speg.) Parodi**. ARGENTINA. Chubut: Escalante, Canadon Pilar, Estancia Los Manantiales, 60 km N of Comodoro Rivadavia; 24 December 1938, Eyerdam W. J. s.n., Beetle A.A., Grondona E. (SI). Neuquén: Ruta 237, next to Estancia La Lonja, 28 November 1963, Vallerini J. 376 (SI). Río Negro: Bariloche, río Lemay, 19 February 2010, Catalán P. s.n. (UZ); Pilcaniyeu, 20 km al S de Paso Flores, 10 December 1981, Cabrera A.L. 33017 with Botta S., Ezcurra C. & Kiesling R. (SI). Santa Cruz: Lago Argentino, south shore near Calafate, 10 January 1939, Eyerdam W. J. s.n. with Beetle A.A. & Grondona E. (SI). San Julian and [unreadable], 1915, Carette E. 15 (SI). Catalán, P., no Lemey, 19 febr 2010, (UZ)**. *Festuca asperella* E.B. Alexeev.** MEXICO. México: Along Hwy. 95, south of Mexico City, just S of El Gordo, 21 August 1972, Dziekanowski C. T, Dunn D. B. & Bolingbroke 2022 (MO, isotype). ***Festuca breviglumis* Swallen.** MEXICO. Jalisco: 11.7 mi SW of Ciudad Guzman, 5 October 2001, Peterson P.M. & Rosales O. 16078 (US); 6.3 mi SW of Mazamitla along Hwy 110 towards Colima, 7 October 2001, Peterson P. M. & Rosales O. 16117 (US)**. *Festuca caldasii* (Kunth) Kunth.** ECUADOR. Chimborazo: Sipambe - Huigra ca km 10, 16 June 1999, Laegaard S. 20405 (AAU). Loja: Catacocha - La Toma km 28, 29 April 1992, Laegaard S. 102535 (AAU); Km 6 above Jimbura along Zumba road, 27 March 1994, Laegaard S. 105256 (AAU); Catamayo, Chinchas-Tambara, fecha, colectores Moreno-Aguilar M. F. F98_iii, Medina I. & Cango G. (HUTPL). ***Festuca carrascana* Stančík & Renvoize.** BOLIVIA. Cochabamba: José Carrasco Torrico, 10-15 km south of Totora along the road to Aiquile, 1 March 2005, Wood J. R. I. 21684 & Haigh A. (LPB, isotype). ***Festuca chiriquensis* Swallen.** PANAMA. Chiriqui: Chiriqui volcano, 29-30 September 1911, Hitchcock A. S. 8197 (US, holotype). ***Festuca chuquisacae* Stančík & Renvoize.** BOLIVIA. Chuquisaca: Tipoyo, cerro Obispo-San Juan, 24 April 1994, Wood J. R. I. 8337 (LPB, isotype). ***Festuca dentiflora* E.B. Alexeev ex Stančík & P.M. Peterson.** PERU. Huanuco: Baños, 1838, Wilkes Explor. Exped. 5 (US, holotype). ***Festuca dichoclada* Pilg.** PERU. Ancash: Huascarán National park, Quebrada Llaca, 24 May 1985, Smith N. 10782 (AAU). Cajamarca: Sais, José Carlos Mariátegui, km 20-40 on Suchubamba-San Juan road, 5 June 1984, Smith D. N. 7534 & Sánchez-Vega I. (MO). ***Festuca fimbriata* Nees.** ARGENTINA. Corrientes: Santo Tome, Arroyo Chimiray and Ruta 40, 8 October 1976, Quarin 3409 (CTES); Garruchos, 22 October 1954, Buikart A. 19659 (BAA, SI); estancia "Garruchos", 1 November 1950 Pedersen T. M. 819 (C, K, MO); Ruta 37, 5 km E of Governador Virasoro, 14 November 1974, Schini A. & Carnevali R. C. 10523 (SI, US); Timbo, costa Río Uruguay, 26 km SE of Colina Garabi, 16 September 1980, Ahumado & Schinini 4085 (CTES). Misiones: Apóstoles, Est. Agrotécnica, October 1977, Cabrera A. 28766, Botta S. M., Kiesling R., Rotman A. D., Tur N. & Zuloaga F. O. (SI); Ruta Prov. 40 and Ayo. Chimiray, 16 October 2000, Zuloaga F. O. & Morrone O. 7155 (SI); Capital, Ruta provinc. no. 1, 12 km S of Posadas, 16 November 1974, Schinini & Carnevali 10687 (CTES). ***Festuca horridula* Pilg.** PERU. Huancavelica: Huillca-orocon, 5 km NW of Conayca, 16 April 1955, Tovar O. 2431 (US). Lima: Salta Cuna, March 1943, Soukup J. 1944 (US); Huarochiri, 107 km W of Lima on Hwy 20 towards La Oroyo [Oroya] at Chicla, 5 April 1997, Peterson P. M. & Tovar O. 14019 (MO). ***Festuca jaliscana* E.B. Alexeev.** MEXICO. Jalisco: NE slopes of the Nevado de Colima, below Canoa de Leoncito, Barranca de la Rosa, 10 October 1952, McVaugh R. 13409 (US, holotype). ***Festuca lugens* (E. Fourn.) Hitchc. ex Hern.-Xol.** MEXICO. Oaxaca: Cumbre de Estepa, September 1842,

Liebmann F. M. 6247, 502 (C, type). ***Festuca quadridentata* Kunth.** ECUADOR. Cañar: S of El Tambo ca 1.5 - 3 km along road to Carshao, 10 June 1999, Laegaard S. 20287 & Sklenář P. (AAU). Chimborazo: Near Alao, along Río Alao, 21 September 1985, Laegaard S. 55290 (AAU); *idem*, Llactapamba, 1 March 1944, Acosta-Solis M. 7581 (US). Chimborazo/Morona Santiago: Hda. Huargualla - Hda. San Eduardo, way to NP Sangay, 20 July 1999 (AAU). ***Festuca steinbachii* E.B. Alexeev.** BOLIVIA. Cochabamba, Cejawald bei "La Aduana", 7 March 1929, Steinbach J. 9533 (LIL, isotype). ***Festuca superba* Parodi ex Türpe.** ARGENTINA. Jujuy: Lagunas de Yala, 30 January 1975, Zuloaga F. O. 383 & Deginani N. B. (SI); *idem*,16 February 1987, Nicora E. 8740, Gómez-Sosa E., Palasi C., Pensiero J. & Rúgolo Z. (SI); *idem*, 16 January 1988, Zuloaga F. O. 3545 & N. B. Deginani (SI); slope of Yala, 29 March 1992, Cabrera A. L. 34886, Deginani A. M., Tur N. & Ulibarri E. (SI); quebrada de Yala, 19 January 1976, Cabrera A. L. 27380, S. Arroyo, N. Bacigalupo, E. Nicora, E. Ulibarri (SI); from San Salvador de Jujuy to Termas de Reyes, 05 March 1987, Zuloaga F. O. 3008, Morrone O. (SI); Termas de Reyes, 5 February 1943, Parodi L. R. 14552 (BAA); *idem*, 07 January 1968, Cabrera A. L. 18879, Deferrari A. M., Frangi J., Marrone M. T., Petriella B. (SI); *idem*, entrada al Balneario, 25 November 2008, Catalan P. 360.08 & Müller J. ( UZ); Yala, Laguna Rodeo, 24 November 2008, Catalan P. 356.08 & Müller J. (UZ). ***Festuca tancitaroensis* Gonz.-Led. & S.D. Koch.** MEXICO. Michoacan: Mt. Tancitaro, 25 July 1941, Leavenworth W.C. & Leavenworth Mrs. W.C. 1213 (US, holotype). ***Festuca urubambana* Stančík.** PERU. Cuzco: Urubamba, lower end of Quebrada Pumahuanca, a deep side-valley of R. Urubamba ca. 2-4km NW of Urubamba, 31 December 1962, Iltis H. H.-C. M. 853 & Ugent D. – V. (K, holotype). ***Festuca valdesii* Gonz.-Led. & S.D. Koch.** MEXICO. Coahuila de Zaragoza: Arteaga, Sierra Zapalinamé, 19 May 1990, Hinton G.S. 20278 & al. (IEB, isotype). ***Festuca woodii* Stančík.** COLOMBIA. Boyaca: Sierra Nevada del Cocuy, hacienda La Esperanza, 29 December 1985, Wood J. 5254 (COL, holotype). ***Festuca venezuelana* Stančík.** VENEZUELA. Tachira: La Grita, La Negra, cross of the two roads to La grita and Pogonero, 10-11 November 2000, Stančík D. 4262 (AAU, isotype).

**Supplementary Table S1.** Genome proportion of repeats estimated by Repeat Explorer2 for individual Loliinae samples (estimated percentages per holoploid genome, 1C). Values in bold correspond to new data generated in this study.

| Loliinae taxon | Class I/LTR/Ty1_copia | | | | | | | | Class I/LTR/Ty3_gypsy | | | | | | | | Class II/TIR | | | | | | | | | | | | | |
|---|---|---|---|---|---|---|---|---|---|---|---|---|---|---|---|---|---|---|---|---|---|---|---|---|---|---|---|---|---|
| | Ale | Angela | Ikeros | Ivana | SIRE | TAR | Tork | OTA | Athila | Tat | Ogre | Tekay | Retand | Reina | CRM | chromovirus | MuDR_ Mutator | EnSpm_ CACTA | hAT | PIF_ Harbinger | Ty1_ copia | Ty3_ gypsy | LTR | LINE | satellite | rDNA (5S-45S) | Mobile element | Repeat (conflicting evidences) | Unclassified (No evidence) | Total |
| **Broad Leaved (BL) Loliinae** | | | | | | | | | | | | | | | | | | | | | | | | | | | | | | |
| *Festuca asperella* | **0.00** | **9.47** | **0.00** | **0.21** | **1.01** | **0.42** | **0.01** | **0.00** | **0.06** | **0.00** | **0.00** | **3.93** | **6.14** | **0.00** | **0.21** | **0.02** | **0.24** | **0.49** | **0.00** | **0.00** | **0.79** | **0.00** | **8.16** | **0.00** | **0.27** | **0.19** | **0.00** | **0.00** | **20.38** | **52.000** |
| *Festuca breviglumis* | **0.00** | **16.98** | **0.00** | **0.71** | **0.41** | **0.34** | **0.00** | **0.00** | **0.00** | **0.00** | **0.01** | **2.68** | **18.65** | **0.00** | **0.02** | **0.00** | **0.20** | **0.65** | **0.00** | **0.00** | **0.00** | **0.00** | **5.59** | **0.00** | **1.37** | **0.02** | **0.00** | **1.51** | **6.47** | **55.610** |
| *Festuca chiriquensis* | **0.00** | **38.63** | **0.00** | **0.30** | **0.86** | **0.27** | **0.09** | **0.00** | **0.53** | **0.00** | **0.00** | **6.36** | **6.76** | **0.00** | **0.22** | **0.00** | **0.32** | **0.58** | **0.02** | **0.00** | **0.00** | **0.40** | **6.82** | **0.00** | **0.64** | **0.15** | **0.00** | **0.02** | **4.53** | **67.500** |
| *Festuca caldasii* | 0.03 | 27.45 | 0.00 | 0.33 | 0.82 | 0.48 | 0.02 | 0.00 | 0.26 | 0.00 | 0.13 | 7.32 | 6.03 | 0.00 | 0.17 | 0.00 | 0.11 | 0.49 | 0.00 | 0.00 | 0.00 | 0.00 | 9.64 | 0.00 | 0.88 | 0.06 | 0.00 | 0.00 | 7.40 | 61.597 |
| *Festuca superba* | 0.03 | 21.07 | 0.00 | 0.90 | 0.68 | 0.49 | 0.01 | 0.00 | 0.00 | 0.00 | 0.01 | 1.54 | 20.31 | 0.00 | 0.05 | 0.00 | 0.51 | 0.92 | 0.00 | 0.00 | 0.00 | 0.00 | 11.01 | 0.00 | 1.40 | 0.26 | 0.00 | 0.00 | 5.71 | 64.879 |
| *Festuca venezuelana* | **0.00** | **15.44** | **0.00** | **0.14** | **0.38** | **0.15** | **0.00** | **0.00** | **0.00** | **0.00** | **0.00** | **4.94** | **4.85** | **0.00** | **0.06** | **0.00** | **0.05** | **0.09** | **0.00** | **0.00** | **0.31** | **0.00** | **10.50** | **0.00** | **0.90** | **0.25** | **0.00** | **0.00** | **26.22** | **64.280** |
| *Festuca dichoclada* | **0.00** | **7.89** | **0.00** | **0.59** | **1.62** | **0.71** | **0.03** | **0.00** | **0.04** | **0.00** | **0.00** | **1.30** | **13.81** | **0.00** | **2.01** | **0.00** | **1.73** | **2.70** | **0.05** | **0.00** | **0.00** | **0.00** | **7.12** | **0.00** | **1.61** | **0.15** | **0.00** | **1.16** | **8.75** | **51.270** |
| *Festuca horridula* | **0.00** | **8.97** | **0.01** | **0.80** | **1.06** | **0.19** | **0.00** | **0.00** | **0.33** | **0.00** | **0.28** | **2.89** | **13.92** | **0.00** | **1.83** | **0.00** | **1.27** | **0.72** | **0.02** | **0.05** | **0.05** | **0.00** | **8.02** | **0.05** | **3.51** | **0.35** | **0.00** | **0.02** | **6.83** | **51.170** |
| *Festuca quadridentata* | 0.00 | 8.56 | 0.01 | 0.35 | 1.64 | 0.25 | 0.00 | 0.00 | 0.43 | 0.00 | 0.10 | 1.75 | 5.83 | 0.00 | 1.88 | 0.00 | 0.10 | 0.69 | 0.00 | 0.00 | 0.00 | 0.00 | 2.37 | 0.09 | 2.41 | 0.27 | 0.00 | 0.00 | 22.24 | 48.970 |
| *Festuca amplissima* | 0.00 | 12.43 | 0.05 | 0.11 | 3.04 | 0.71 | 0.23 | 0.00 | 0.26 | 0.00 | 0.02 | 2.05 | 7.87 | 0.00 | 0.67 | 0.00 | 0.79 | 1.54 | 0.00 | 0.00 | 0.00 | 0.00 | 6.89 | 0.16 | 2.72 | 0.12 | 0.00 | 0.00 | 12.58 | 52.254 |
| *Festuca valdesii* | **0.00** | **11.38** | **0.00** | **0.75** | **1.82** | **0.66** | **0.06** | **0.00** | **0.22** | **0.00** | **0.00** | **1.39** | **9.65** | **0.00** | **3.40** | **0.00** | **0.24** | **0.68** | **0.00** | **0.00** | **0.00** | **0.00** | **11.07** | **0.04** | **2.94** | **0.41** | **0.00** | **1.04** | **10.17** | **55.920** |
| *Festuca argentina* | **0.00** | **6.77** | **0.04** | **0.41** | **8.84** | **0.70** | **0.05** | **0.00** | **0.43** | **0.00** | **0.20** | **3.23** | **16.33** | **0.00** | **1.41** | **0.00** | **0.75** | **2.33** | **0.00** | **0.00** | **0.00** | **0.00** | **4.67** | **0.10** | **2.66** | **0.29** | **0.00** | **1.45** | **6.04** | **56.700** |
| *Festuca kingii* | **0.11** | **6.72** | **0.01** | **0.37** | **1.42** | **0.26** | **0.00** | **0.00** | **0.04** | **0.00** | **0.00** | **0.65** | **12.77** | **0.00** | **0.27** | **0.00** | **0.27** | **0.63** | **0.00** | **0.00** | **0.00** | **0.00** | **6.37** | **0.01** | **4.72** | **0.45** | **0.00** | **0.00** | **18.67** | **53.630** |
| *Festuca spectabilis* | 0.00 | 8.94 | 0.00 | 0.00 | 2.47 | 0.73 | 0.10 | 0.00 | 0.07 | 0.00 | 0.12 | 3.54 | 10.48 | 0.00 | 2.38 | 0.00 | 0.35 | 4.13 | 0.00 | 0.05 | 0.00 | 0.00 | 7.32 | 0.20 | 2.04 | 0.77 | 0.00 | 0.00 | 7.71 | 51.406 |
| *Festuca africana* | 0.00 | 8.42 | 0.00 | 0.00 | 0.46 | 0.06 | 0.00 | 0.00 | 0.30 | 0.00 | 4.63 | 4.75 | 1.32 | 0.00 | 0.14 | 0.00 | 0.13 | 1.14 | 0.00 | 0.00 | 0.00 | 0.00 | 5.74 | 0.00 | 1.70 | 0.04 | 0.00 | 0.00 | 32.26 | 61.096 |
| *Festuca mekiste* | 0.00 | 8.79 | 0.00 | 0.01 | 3.08 | 0.24 | 0.00 | 0.00 | 2.77 | 0.00 | 7.92 | 11.14 | 1.91 | 0.00 | 0.35 | 0.00 | 0.27 | 2.86 | 0.00 | 0.00 | 0.00 | 0.00 | 2.68 | 0.00 | 3.01 | 0.03 | 0.00 | 0.00 | 6.50 | 51.573 |
| *Festuca durandoi* | 0.00 | 6.81 | 0.00 | 0.00 | 3.60 | 0.47 | 0.00 | 0.00 | 0.17 | 0.00 | 0.16 | 3.87 | 18.20 | 0.00 | 1.84 | 0.00 | 0.19 | 4.04 | 0.00 | 0.09 | 0.00 | 0.00 | 4.90 | 0.02 | 0.96 | 0.10 | 0.00 | 0.87 | 7.81 | 54.121 |
| *Festuca paniculata* | 0.00 | 7.51 | 0.00 | 0.00 | 3.75 | 0.43 | 0.02 | 0.00 | 0.45 | 0.00 | 0.00 | 2.30 | 14.83 | 0.00 | 0.81 | 0.00 | 0.02 | 0.51 | 0.00 | 0.03 | 0.00 | 0.00 | 5.82 | 0.03 | 0.82 | 0.89 | 2.77 | 0.00 | 13.16 | 54.140 |
| *Festuca lasto* | 0.00 | 11.83 | 0.09 | 0.00 | 2.73 | 0.59 | 0.02 | 0.00 | 3.42 | 0.00 | 0.00 | 8.51 | 6.85 | 0.00 | 1.76 | 0.00 | 0.03 | 1.72 | 0.00 | 0.00 | 0.00 | 0.00 | 1.76 | 0.01 | 0.84 | 0.17 | 0.00 | 0.00 | 14.11 | 54.461 |
| *Festuca triflora* | 0.00 | 15.24 | 0.00 | 0.00 | 1.34 | 0.33 | 0.14 | 0.00 | 5.98 | 0.00 | 0.11 | 7.71 | 7.15 | 0.00 | 0.78 | 0.00 | 0.13 | 0.85 | 0.00 | 0.00 | 0.00 | 0.00 | 0.00 | 0.00 | 1.78 | 0.51 | 0.00 | 0.00 | 14.93 | 56.981 |

| | | | | | | | | | | | | | | | | | | | | | | | | | | | | | |
|---|---|---|---|---|---|---|---|---|---|---|---|---|---|---|---|---|---|---|---|---|---|---|---|---|---|---|---|---|---|
| *Festuca pratensis* | 0.04 | 5.41 | 0.01 | 0.00 | 3.77 | 0.19 | 0.00 | 0.00 | 7.18 | 0.00 | 0.66 | 4.89 | 14.88 | 0.00 | 4.26 | 0.00 | 0.01 | 2.02 | 0.00 | 0.00 | 0.00 | 0.00 | 2.17 | 0.01 | 1.81 | 0.69 | 0.00 | 0.40 | 10.29 | 58.684 |
| *Festuca atlantigena* | 0.00 | 2.84 | 0.02 | 0.00 | 0.60 | 0.08 | 0.01 | 0.00 | 0.14 | 0.00 | 0.00 | 7.50 | 11.16 | 0.00 | 1.49 | 0.00 | 0.06 | 1.62 | 0.00 | 0.03 | 0.00 | 0.00 | 6.42 | 0.08 | 2.13 | 0.37 | 0.00 | 0.09 | 11.43 | 46.088 |
| *Festuca molokaiensis* | 0.00 | 5.94 | 0.03 | 1.26 | 9.96 | 0.03 | 0.00 | 0.00 | 0.01 | 0.00 | 1.55 | 5.71 | 21.35 | 0.00 | 0.26 | 0.00 | 1.37 | 4.95 | 0.00 | 0.00 | 0.00 | 0.00 | 1.49 | 0.02 | 1.12 | 0.25 | 0.00 | 1.49 | 7.09 | 63.853 |
| Fine leaved (FL) Loliinae | | | | | | | | | | | | | | | | | | | | | | | | | | | | | | |
| *Festuca fimbriata* | 0.01 | 4.47 | 0.15 | 0.04 | 1.60 | 1.18 | 0.12 | 0.00 | 0.04 | 0.00 | 0.34 | 0.21 | 1.66 | 0.00 | 0.09 | 0.00 | 0.20 | 1.18 | 0.00 | 0.00 | 0.43 | 0.00 | 1.62 | 0.02 | 3.91 | 0.05 | 0.00 | 0.00 | 21.54 | 38.855 |
| *Festuca asplundii* | 0.00 | 9.97 | 0.52 | 0.00 | 8.17 | 0.53 | 0.13 | 0.00 | 1.31 | 0.00 | 0.82 | 0.02 | 1.96 | 0.00 | 0.67 | 0.00 | 0.93 | 5.52 | 0.00 | 0.01 | 0.00 | 0.00 | 3.27 | 0.00 | 1.83 | 0.04 | 0.00 | 0.97 | 11.75 | 48.412 |
| *Festuca procera* | 0.00 | 10.88 | 0.36 | 0.00 | 7.10 | 0.60 | 0.12 | 0.01 | 0.87 | 0.00 | 0.34 | 0.21 | 4.32 | 0.00 | 1.48 | 0.00 | 0.80 | 4.67 | 0.00 | 0.00 | 0.00 | 0.00 | 0.00 | 0.00 | 2.66 | 0.05 | 0.00 | 2.09 | 11.66 | 48.212 |
| *Festuca chimborazensis* | 0.00 | 10.89 | 0.06 | 0.02 | 6.98 | 0.67 | 0.00 | 0.01 | 1.06 | 0.00 | 0.06 | 0.00 | 4.17 | 0.00 | 1.16 | 0.00 | 0.65 | 5.04 | 0.00 | 0.09 | 0.00 | 0.00 | 2.15 | 0.00 | 4.09 | 0.30 | 0.00 | 1.07 | 8.99 | 47.449 |
| *Festuca holubii* | 0.00 | 10.87 | 0.07 | 0.00 | 6.86 | 0.76 | 0.00 | 0.00 | 0.61 | 0.00 | 0.02 | 0.01 | 3.52 | 0.00 | 1.10 | 0.00 | 0.54 | 4.54 | 0.00 | 0.01 | 0.00 | 0.00 | 0.90 | 0.01 | 5.96 | 0.42 | 0.00 | 0.99 | 13.32 | 50.518 |
| *Festuca pampeana* | 0.00 | 0.52 | 0.00 | 0.06 | 0.63 | 0.10 | 0.06 | 0.00 | 0.00 | 0.00 | 0.33 | 0.00 | 6.47 | 0.00 | 0.03 | 0.00 | 0.19 | 0.85 | 0.00 | 0.00 | 0.00 | 0.00 | 2.85 | 0.00 | 4.77 | 1.02 | 0.00 | 0.00 | 23.59 | 41.481 |
| *Festuca gracillima* | 0.00 | 4.50 | 0.15 | 0.02 | 1.74 | 0.61 | 0.00 | 0.00 | 0.87 | 0.00 | 1.22 | 0.01 | 6.05 | 0.00 | 1.54 | 0.00 | 0.92 | 0.76 | 0.00 | 0.00 | 0.00 | 0.00 | 8.23 | 0.00 | 1.45 | 0.61 | 0.00 | 6.04 | 13.95 | 48.684 |
| *Festuca abyssinica* | 0.00 | 3.65 | 0.00 | 0.07 | 0.96 | 0.08 | 0.00 | 0.00 | 0.15 | 0.00 | 1.85 | 1.78 | 3.83 | 0.00 | 0.34 | 0.00 | 0.41 | 1.56 | 0.00 | 0.00 | 0.00 | 0.00 | 3.45 | 0.00 | 4.93 | 0.31 | 0.00 | 0.00 | 26.92 | 50.267 |
| *Festuca rubra* | 0.00 | 0.08 | 0.00 | 0.00 | 0.96 | 0.30 | 0.01 | 0.00 | 8.55 | 0.00 | 0.61 | 1.24 | 2.11 | 0.00 | 0.69 | 0.00 | 0.01 | 0.95 | 0.00 | 0.00 | 0.00 | 0.00 | 0.76 | 0.00 | 6.56 | 0.70 | 0.00 | 0.32 | 22.79 | 46.646 |
| *Festuca ovina* | 0.03 | 0.26 | 0.02 | 0.00 | 3.16 | 0.33 | 0.00 | 0.00 | 7.18 | 0.00 | 0.59 | 2.04 | 4.26 | 0.00 | 1.04 | 0.00 | 0.00 | 2.01 | 0.00 | 0.01 | 0.00 | 0.00 | 2.75 | 0.03 | 5.22 | 0.28 | 0.00 | 7.10 | 12.28 | 48.550 |
| *Festuca capillifolia* | 0.00 | 0.75 | 0.00 | 0.00 | 2.32 | 0.14 | 0.01 | 0.00 | 1.58 | 0.00 | 0.22 | 4.87 | 4.03 | 0.00 | 1.41 | 0.00 | 0.00 | 1.86 | 0.00 | 0.00 | 0.00 | 0.00 | 9.77 | 0.00 | 5.16 | 0.65 | 0.00 | 0.00 | 24.23 | 57.019 |
| *Vulpia ciliata* | 0.14 | 3.81 | 0.00 | 0.00 | 0.78 | 0.49 | 0.16 | 0.01 | 0.22 | 0.00 | 0.08 | 0.56 | 16.79 | 0.00 | 0.86 | 0.00 | 0.11 | 1.60 | 0.00 | 0.00 | 0.00 | 0.00 | 8.45 | 0.33 | 2.76 | 0.70 | 0.00 | 2.33 | 11.74 | 51.766 |
| *Festuca gautieri* | **0.00** | **4.85** | **0.04** | **0.11** | **4.41** | **0.41** | **0.00** | **0.00** | **0.14** | **0.00** | **0.21** | **1.25** | **13.15** | **0.00** | **1.09** | **0.00** | **0.21** | **2.14** | **0.00** | **0.04** | **0.00** | **0.00** | **7.35** | **0.02** | **1.35** | **0.86** | **0.00** | **0.00** | **10.71** | **48.340** |
